# Class 1 integrons and multiple mobile genetic elements in clinical isolates of the *Klebsiella pneumoniae* complex from a tertiary hospital in eastern China

**DOI:** 10.3389/fmicb.2023.985102

**Published:** 2023-03-06

**Authors:** Lan Wang, Mei Zhu, Chunxia Yan, Yanfang Zhang, Xuying He, Lin Wu, Jiefeng Xu, Junwan Lu, Qiyu Bao, Yunliang Hu, Teng Xu, Jialei Liang

**Affiliations:** ^1^Medical Molecular Biology Laboratory, School of Medicine, Jinhua Polytechnic, Jinhua, China; ^2^Department of Clinical Laboratory, Zhejiang Hospital, Hangzhou, Zhejiang, China; ^3^The Second Affiliated Hospital and Yuying Children’s Hospital, Wenzhou Medical University, Wenzhou, China; ^4^Institute of Translational Medicine, Baotou Central Hospital, Baotou, China

**Keywords:** *Klebsiella* species identification, multilocus sequence typing, integron, antimicrobial resistance, mobile genetic element

## Abstract

**Background:**

The emergence of highly drug-resistant *K. pneumoniae*, has become a major public health challenge. In this work, we aim to investigate the diversity of species and sequence types (STs) of clinical *Klebsiella* isolates and to characterize the prevalence and structure of class 1 integrons.

**Methods:**

Based on the whole genome sequencing, species identification was performed by 16S rRNA gene homology and average nucleotide identity (ANI) analysis. STs were determined in accordance with the international MLST schemes for *K. pneumoniae* and *K. variicola*. Integron characterization and comparative genomic analysis were performed using various bioinformatic tools.

**Results:**

Species identification showed that the 167 isolates belonged to four species: *K. pneumoniae*, *K. variicola* subsp. *variicola*, *K. quasipneumoniae* and *K. aerogenes*. Thirty-six known and 5 novel STs were identified in *K. pneumoniae*, and 10 novel STs were identified in *K. variicola* subsp. *variicola*. Class 1 integrons were found in 57.49% (96/167) of the isolates, and a total of 169 resistance gene cassettes encoding 19 types of resistance genes, including carbapenem resistance gene (*bla*_IPM-4_) and class D β-lactamases gene (*bla*_OXA-1_ and *bla*_OXA-10_), were identified. Among the 17 complete genomes, 29 class 1 integrons from 12 groups were found, only 1 group was encoded on chromosomes. Interestingly, one plasmid (pKP167-261) carrying two copies of approximately 19-kb IS*26*-Int1 complex resistance region that contains an integron and a multidrug resistance gene fragment.

**Conclusion:**

The results of this work demonstrated that the species and STs of the clinical *Klebsiella* isolates were more complex by the whole genome sequence analysis than by the traditional laboratory methods. Finding of the new structure of MGEs related to the resistance genes indicates the great importance of deeply exploring the molecular mechanisms of bacterial multidrug resistance.

## Introduction

Since all members of the *Klebsiella pneumoniae* species complex (KpSC) show overlapping biochemical and phenotypic characteristics, classification of the genomic features of clinical isolates identified as *K. pneumoniae* based on biochemical assays or mass spectrometry (MALDI-TOF) could result in misclassification (Long et al., 2017; Rodriguez-Medina et al., 2019). Whole-genome sequencing (WGS) has clarified that these multiple related species and subspecies share 95–96% average nucleotide identity (ANI) with *K. pneumoniae* but only 90% ANI with other *Klebsiella* species (Wyres et al., 2020; Lam et al., 2021). *K. pneumoniae* (Kp1) is very common in clinical collections and usually accounts for approximately 85% of the isolates identified as *K. pneumoniae*. *K. variicola* and *K. quasipneumoniae* are relatively common pathogens in hospital-acquired infections (10–20% of the incidence of *K. pneumoniae*; Holt et al., 2015). Along with several other bacteria, *K. pneumoniae* has shown a dramatic increase in antibiotic resistance in recent decades (Paczosa and Mecsas, 2016; Navon-Venezia et al., 2017; Effah et al., 2020). Through horizontal gene transfer mediated by plasmid and mobile genetic elements (MGEs), more than 400 antimicrobial resistance genes were found in *K. pneumoniae* (Navon-Venezia et al., 2017; Wyres and Holt, 2018). A recent study estimating the current known *K. pneumoniae* “pangenome” demonstrated that the pangenome is “open,” indicating that these species have a high horizontal gene transfer rate (Martin and Bachman, 2018).

Mobile genetic elements, such as insert sequences (ISs), transposons (Tns) and integrons, play an important role in increasing antibiotic resistance (Partridge et al., 2018). Bacteria share MGEs and their associated resistance genes with other bacterial species *via* horizontal gene transfer (HGT), which has promoted the accumulation and dissemination of antibiotic resistance genes (ARGs) in bacteria (Tao et al., 2022). Integrons constitute an important and near-ubiquitous class of genetic elements. An integron is generally defined by the presence of an *int* gene encoding an integrase of the tyrosine recombinase family, an *attI* recombination site and a promoter (Domingues et al., 2015). As classified by the sequence encoding integrase, five classes of integrons associated with drug resistance have been found (Cambray et al., 2010). The typical structure of a class 1 integron is composed of two conserved segments (5′ CS and 3′ CS) and a variable region with one or more antimicrobial resistance gene cassettes (Hall and Collis, 1995; Fluit and Schmitz, 1999). Class 1 integrons are the most common and widespread among clinical gram-negative bacteria, including *Escherichia coli*, *Klebsiella*, *Salmonella*, *Shigella*, *Yersinia* and other disease-causing bacteria, because of their close association with transposons, often embedded within conjugative plasmids (Goldstein et al., 2001; Lima et al., 2014). Class 1 integrons function as a genetic platform for antimicrobial resistance gene cassette capture. At least 200 different gene cassettes have been identified from class 1 integrons, most of which are antibiotic resistance gene cassettes, including the genes conferring resistance to the quaternary ammonium compound family, aminoglycosides, sulfonamides, quinolones, chloramphenicol, fosfomycin, trimethoprim, β-lactams, and other clinically relevant antibiotics (Mazel, 2006; Cambray et al., 2010; Deng et al., 2015).

Recently, the emergence of multidrug-resistant (MDR) *K. pneumoniae* has become a serious issue in healthcare settings worldwide. In China, a close relationship between MDR *K. pneumoniae* strains and the presence of integrons has been demonstrated (Li et al., 2013; Xu et al., 2017; Liao et al., 2020a). Therefore, understanding the molecular characterization of class 1 integrons in *K. pneumoniae* is essential for the implementation of intervention strategies. In this work, we investigated the species and sequence type (ST) diversity and drug resistance profiles of clinical *Klebsiella* isolates and characterized the structure of resistance gene-related class 1 integrons. Notably, we identified a double IS*26*-Int1 complex resistance region in an IncFIB (K) plasmid for the first time.

## Materials and methods

### Sample collection and bacterial identification

A total of 167 clinical *Klebsiella* isolates were collected from patients in different wards in Zhejiang Hospital in Hangzhou, Zhejiang, China, in 2019. Zhejiang Hospital, one of the largest public hospitals in Zhejiang Province, is a Grade III, Class A general hospital integrating medical treatment, teaching, research, prevention and health care. The largest fraction of the specimens were sputum specimens (44.91%, 75/167), followed by urine (19.76%, 33/167), blood (10.18%, 17/167), throat swabs (7.78%, 13/167), feces (4.79%, 8/167), pus (4.19%, 7/167), alveolar lavage fluid (1.80%, 3/167), duodenal drainage (1.80%, 3/167), prostatic fluid (0.60%, 1/167), catheter specimens (0.60%, 1/167), subglottic secretions (0.60%, 1/167), wound secretions (0.60%, 1/167), perianal secretions (0.60%, 1/167), and other secretions (1.80%, 3/167). All of the isolates were initially identified using the Vitek-60 microorganism auto analysis system (BioMerieux Corporate, Craponne, France). Further species identification was performed by 16S rRNA gene homology comparison (Clarridge, 2004) and ANI analysis using FastANI (Jain et al., 2018). According to previous publications, seven strains representative of the KpSC (*K. pneumoniae*: CP003200, *K. quasipneumoniae* subsp. *quasipneumoniae*: AYIC00000000, *K. quasipneumoniae* subsp. *similipneumoniae*: CP084787, *K. quasivariicola*: AKYX00000000, *K. variicola* subsp. *tropica*: CP084767, *K. variicola* subsp. *variicola*: CP072130, and *K. africana*: CP084874) and one reference strain (*K. aerogenes*, FKIV00000000) were selected for ANI analysis (Goris et al., 2007; Wyres et al., 2020).

### Antibiotic susceptibility testing

Minimum inhibitory concentrations (MICs) were determined using the agar dilution method following the guidelines of the Clinical and Laboratory Standards Institute (CLSI), and the susceptibility patterns were interpreted according to the CLSI breakpoint criteria (CLSI, 2019). Multidrug-resistant (MDR) strains were defined as those that were unsusceptible to ≥1 agent in each of >3 antimicrobial categories (Magiorakos et al., 2012). The antimicrobials tested in this work included aminoglycosides (gentamicin and amikacin), cephalosporins (cefepime and ceftazidime), quinolones (nalidixic acid), monobactams (aztreonam), carbapenems (meropenem), phosphonic acids (fosfomycin), tetracyclines (tetracycline), glycylcyclines (tigecycline) and phenicols (chloramphenicol). *Escherichia coli* ATCC 25922 was used as a reference strain for quality control. The interpretive criteria for tigecycline susceptibility (≤2 μg/ml, susceptible; 4 μg/ml, intermediate; ≥8 μg/ml, resistant) were based on the breakpoints established by the Food and Drug Administration^1^.

### Genome sequencing, assembly, annotation and bioinformatic analysis

The whole-genome DNA of 167 *Klebsiella* strains was extracted using the AxyPrep Bacterial Genomic DNA Miniprep kit (Axygen Biosciences, Union City, CA, United States). WGS of 167 isolates was performed using the Illumina HiSeq 2,500, of which the 17 isolates with the widest resistance spectra, the highest MIC levels and the most resistance genes were further sequenced by PacBio RS II platforms by Shanghai Personal Biotechnology Co., Ltd. (Shanghai, China). The Illumina short reads and PacBio long reads were initially assembled by SPAdes v3.14.1, Canu v2.1 and Unicycler v0.8 (Bankevich et al., 2012; Koren et al., 2017; Wick et al., 2017). Further correction was conducted by using Pilon and SAMtools to improve assembly quality by mapping short reads to the draft of the whole-genome assembly (Li et al., 2009; Walker et al., 2014). The open reading frames (ORFs) were predicted and annotated using Prokka v1.14.0 and further annotated by DIAMOND against the UniProtKB/Swiss-Prot and NCBI nonredundant protein databases with an e-value threshold of 1e-5 (Seemann, 2014; Buchfink et al., 2015). Identification of resistance genes was performed using Resistance Gene Identifier (RGI) v4.0.3 in the Comprehensive Antibiotic Resistance Database (CARD; Silva et al., 2011). Identification of ISs and integrons was performed using ISfinder and INTEGRALL, respectively (Siguier et al., 2006; Moura et al., 2009). A phylogenetic tree was inferred from the Mash distances of the 167 whole genome sequences. Pairwise distances were calculated using Mash v2.1.11 and used to infer a phylogenetic tree with iToL (Ondov et al., 2016; Letunic and Bork, 2021). Gview was used to construct basic genomic features^2^. Easyfig was used to generate a figure showing structural comparisons and the nucleotide identities between several segments in a linear fashion (Sullivan et al., 2011). Comparisons of the nucleotide sequences were performed using BLASTN. Molecular types were determined in accordance with the international MLST schemes for *K. pneumoniae* (Diancourt et al., 2005) and *K. variicola* (Barrios-Camacho et al., 2019).

### Statistical analysis

Statistical analysis was performed by Fisher’s exact test using SPSS (version 22.0), and a *p* value of *p* < 0.05 was considered to indicate statistical significance.

## Results and discussion

### Molecular identification of *Klebsiella* isolates

**Figure 1:**
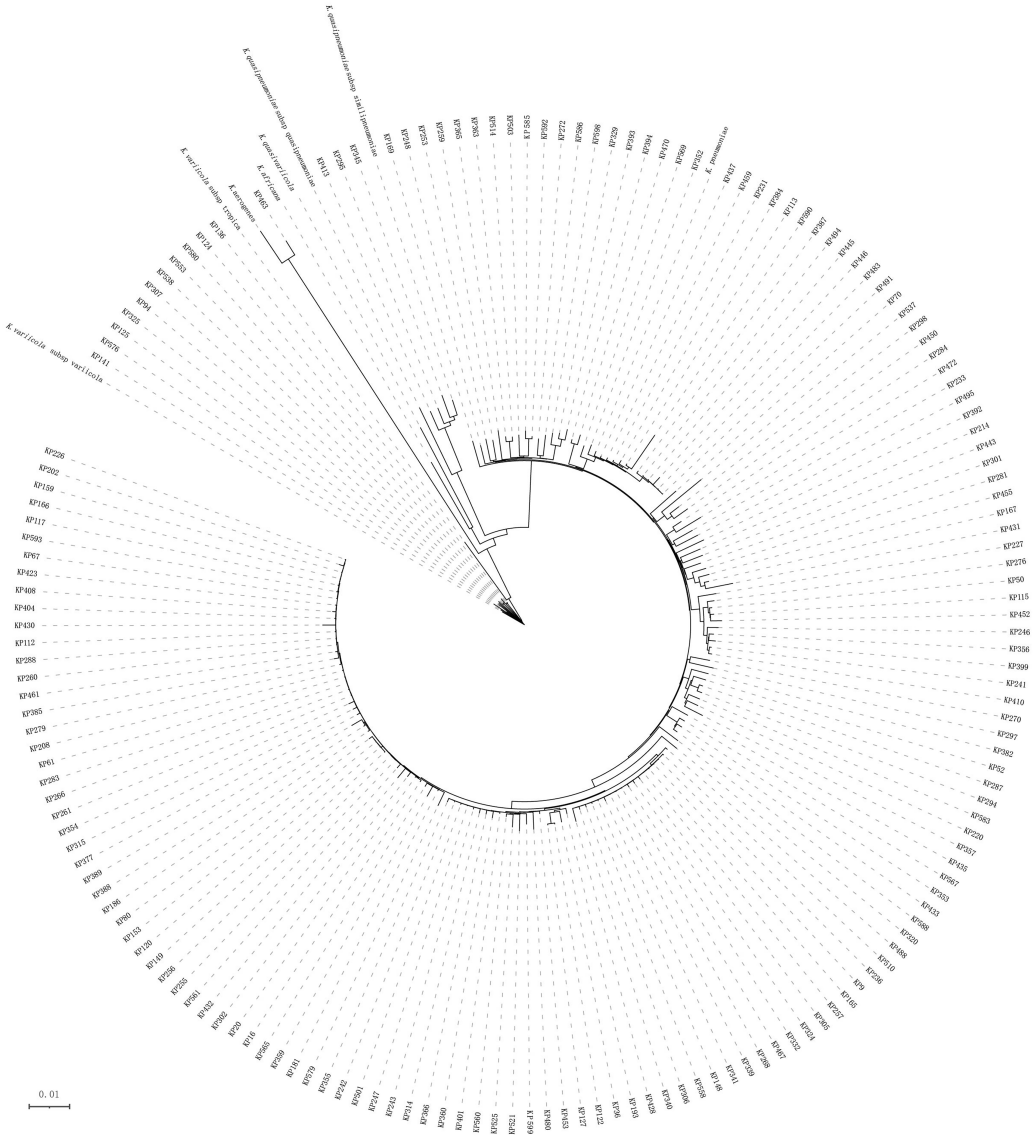
Phylogenetic tree of 167 *Klebsiella* isolates. This whole-genome-based tree shows the phylogenetic relationships between those 167 isolates, their close relatives in the seven *Klebsiella pneumoniae* species complex (*K. pneumoniae*: CP003200, *K. quasipneumoniae* subsp. *quasipneumoniae*: AYIC00000000, *K. quasipneumoniae* subsp. *similipneumoniae*: CP084787, *K. quasivariicola*: AKYX00000000, *K. variicola* subsp. *tropica*: CP084767, *K. variicola* subsp. *variicola*: CP072130, and *K. africana*: CP084874), and *K. aerogenes* (FKIV00000000).

According to the Vitek-60 microorganism auto analysis system, the 167 clinical isolates were all identified as *K. pneumoniae*. Homology analysis of the 16S rRNA gene revealed that the 167 isolates belonged to two species, 166 *K. pneumoniae* isolates and 1 *K. aerogenes* isolate (Supplementary Table S1). However, the results of the ANI analysis of these strains showed that 152, 11, 3 and 1 isolates were *K. pneumoniae*, *K. variicola* subsp. *variicola*, *K. quasipneumoniae* subsp. *similipneumoniae*, and *K. aerogenes*, respectively. They all showed ANIs of more than 98% with the reference strains of the corresponding species (Supplementary Table S2). Whole-genome-based tree showing the phylogenetic relationships between those 167 isolates, their close relatives in the *K. pneumoniae* species complex and *K. aerogenes* (Figure 1).

**Figure 2:**
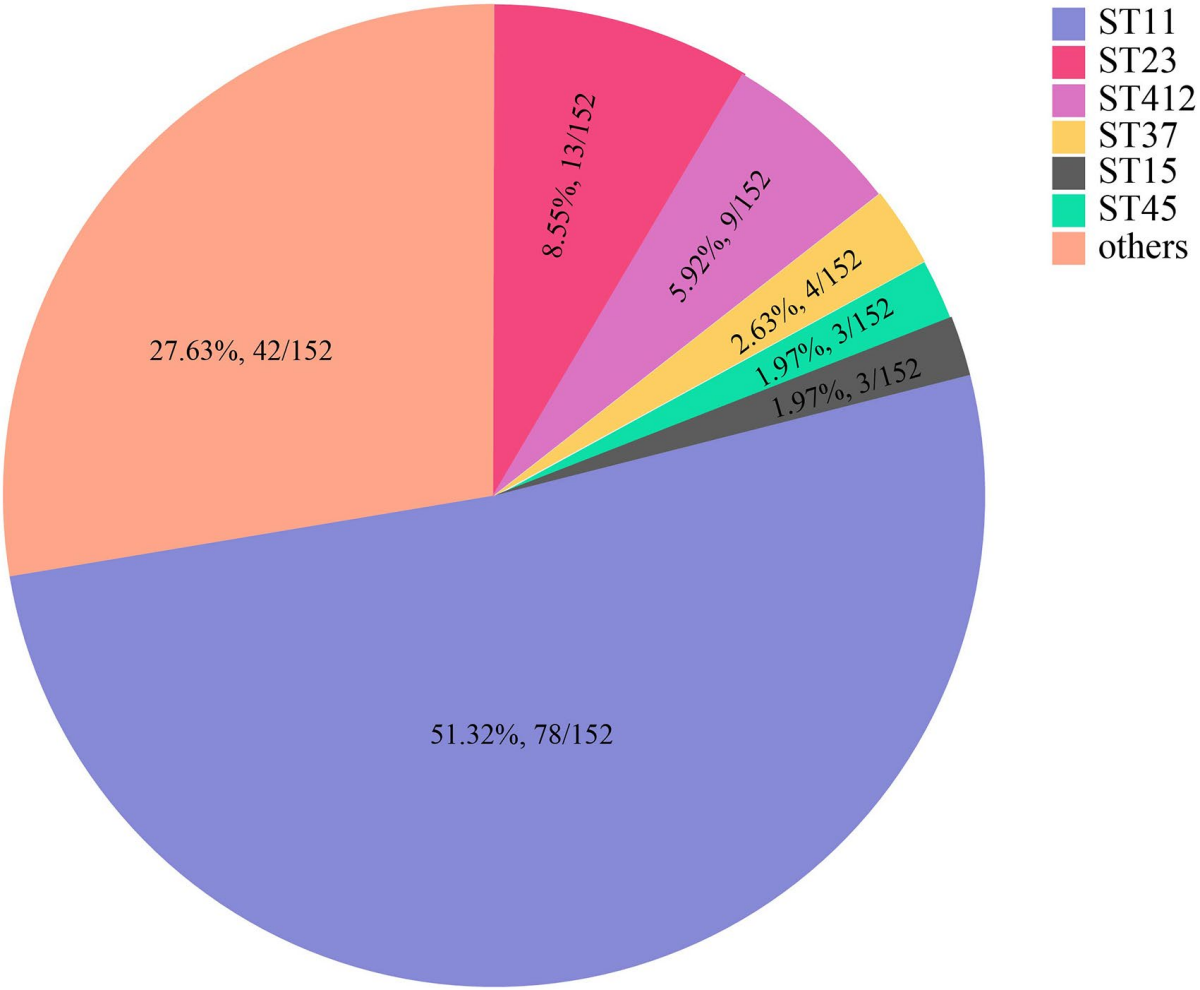
The sequence types (STs) of *K. pneumoniae*. * Only STs with at least three isolates are presented. STs with fewer isolates are included in the category “others.” The detailed ST information is in Supplementary Table S3.

**Figure 3:**
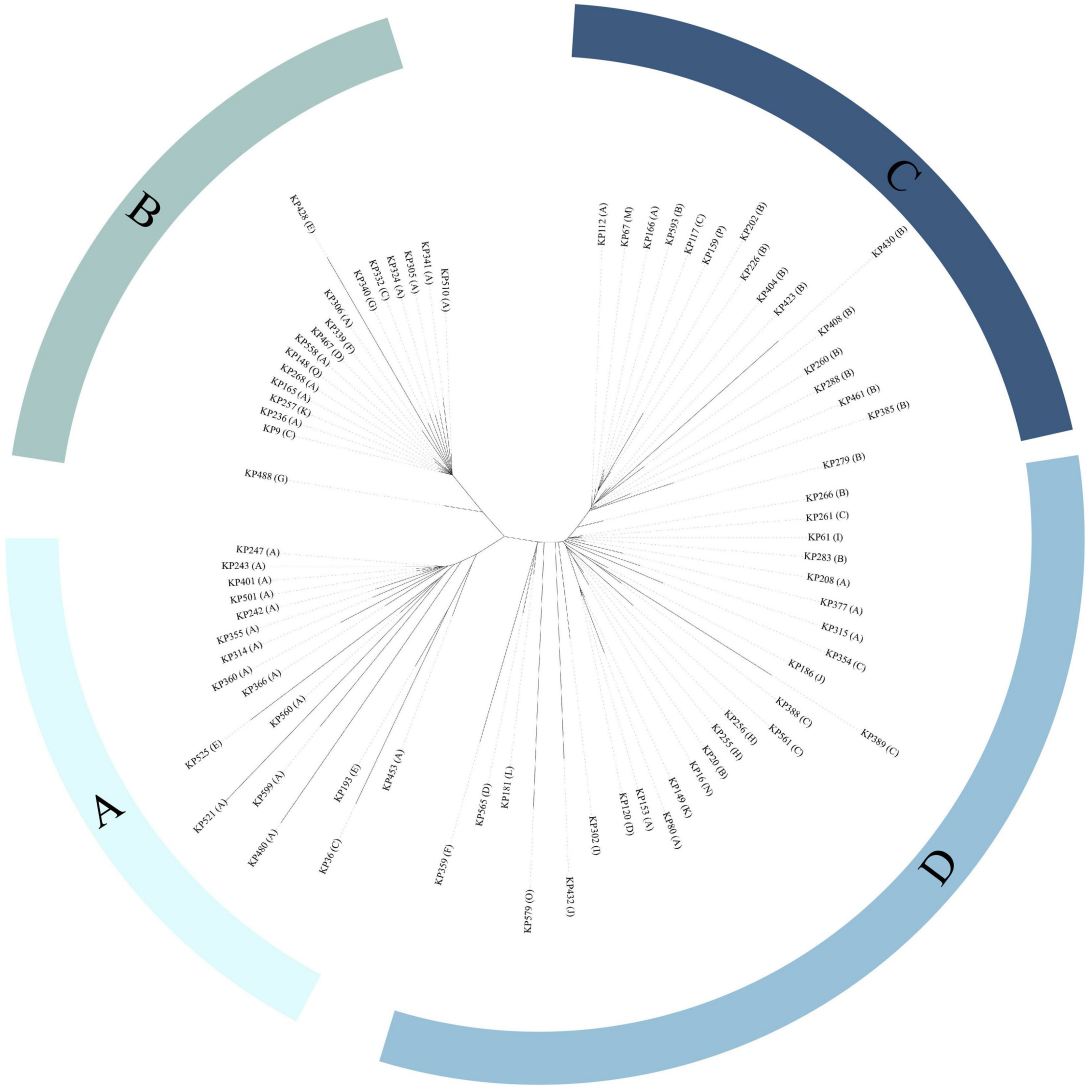
Phylogenetic tree of ST11 *Klebsiella* isolates. Department abbreviations: A, Intensive Care Unit; B, Hematology Department; C, Respiratory Medicine Department; D, Neurointerventional Department; E, Community Hospital; F, Neurosurgery Department; G, General Surgery Department; H, Urology Department; I, Geriatrics Outpatient Clinic; J, Rehabilitation Medicine Department; K, Cardiac Macrovascular Surgery Department; L, Nephrology Department; M, Neurology Department; N, Orthopedics Department; O, Department of Infection Disease; P, Department of Plastic Surgery; Q, Cardiovascular Medicine Department.

MLST analysis revealed that 147 of the 152 *K. pneumoniae* isolates could be assigned to 36 known sequence types (STs). ST11 was the most prevalent, accounting for more than half of the total (51.32%, 78/152), followed by ST23 (8.55%, 13/152) and ST412 (5.92%, 9/152; Figure 2; Supplementary Table S3; Supplementary Figure S1). Five isolates could not be assigned to any of the existing STs. According to the MLST criteria, these isolates represented 5 new STs, designated ST6013 (id: 21814), ST6023 (id: 21835), ST6026 (id: 21838), ST6254 (id: 22343), and ST6255 (id: 22344; Supplementary Table S3). Clonal relatedness analysis of the ST11 strains showed that clonal dissemination occurred among the different departments within the hospital (such as clusters B and D of Figure 3), and clonal outbreaks appeared in some departments, such as the Intensive Care Unit (A) and Hematology Department (B) (clusters A and C of Figure 3). Among the 11 *K. variicola* subsp. *Variicola* isolates, none matched the known ST profiles. We therefore submitted data to the *K. variicola* MLST system and obtained 10 new STs (with one ST represented by two isolates; Table 1).

**Table 1:** The sequence types (STs) of *K. variicola* subsp. *variicola*.

Strain	ST	leuS	pgi	pgk	phoE	pyrG	rpoB	fusA
KP125	333	1	18	12	79	56	33	2
KP124	427	11	1	2	41	1	1	6
KP136	427	11	1	2	41	1	1	6
KP141	428	19	4	5	46	5	7	4
KP307	429	31	60	2	8	58	1	4
KP325	430	9	79	12	8	3	5	2
KP553	431	9	24	3	94	54	1	6
KP538	431	9	24	3	94	54	1	6
KP576	432	93	3	6	32	1	1	4
KP580	433	10	24	44	24	1	1	2
KP94	434	1	36	12	25	3	1	2

### Antibiotic susceptibility and resistance genes

**Figure 4:**
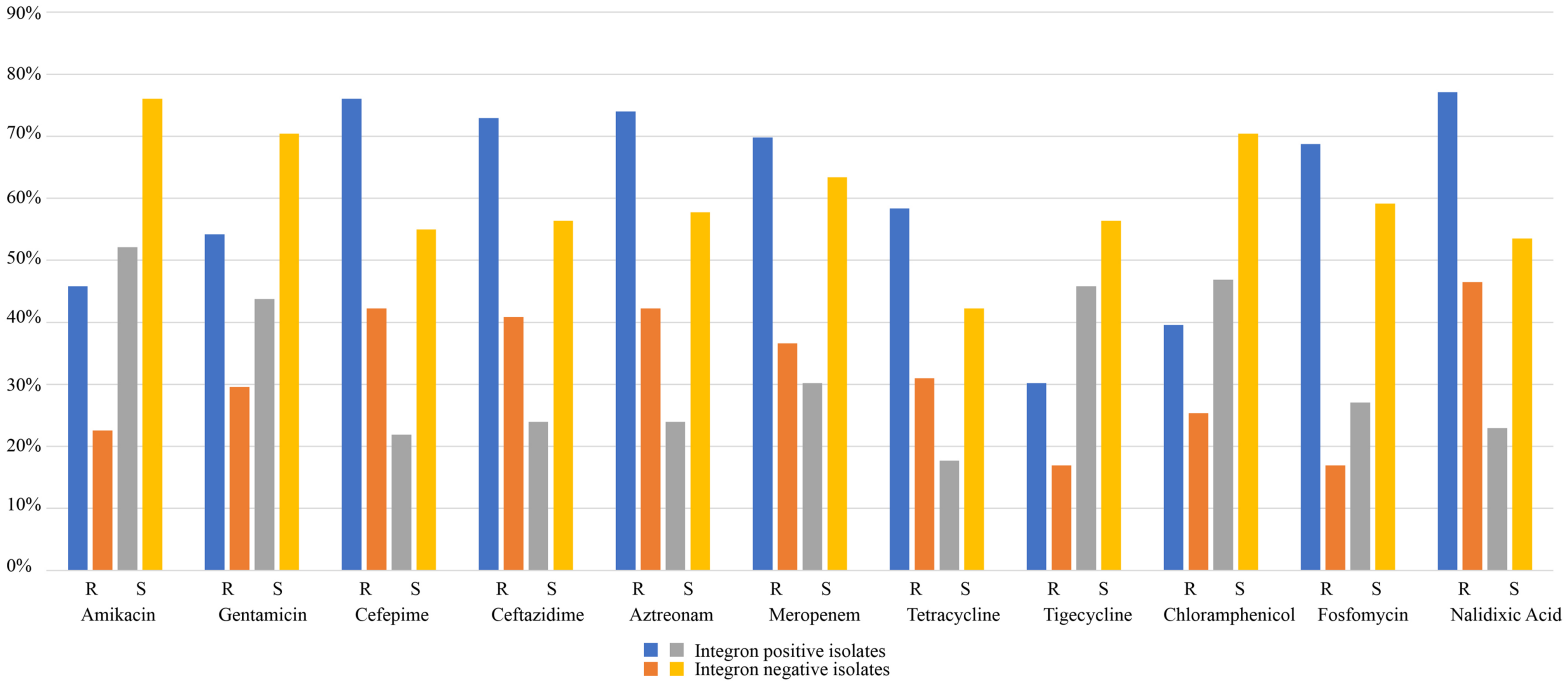
Comparison of the integron-positive and integron-negative isolates in terms of resistance rates against 11 antimicrobials.

Antibiotic susceptibility tests revealed that the 167 isolates had a high prevalence (≥ 50%) of resistance to 5 of the 11 antimicrobials tested, including nalidixic acid (64.07%), cefepime (61.68%), aztreonam (60.48%), ceftazidime (59.28%), and meropenem (55.69%). The prevalence of resistance for the remaining 6 antimicrobials was 46.71% (both fosfomycin and tetracycline), 43.71% (gentamicin), 35.93% (amikacin), 33.53% (chloramphenicol) and 24.55% (tigecycline; Table 2; Supplementary Tables S4, S5; Figure 4).

**Table 2:** Comparison of the integron-positive and integron-negative isolates in terms of the resistance rates against 11 antimicrobials.

Antimicrobial	Integron-positive isolates (*n* = 96)		Integron-negative isolates (*n* = 71)		Total isolates (*n* = 167)		*p* value
	R no. (%)	S no. (%)	R no. (%)	S no. (%)	R no. (%)	S no. (%)	
Amikacin	44 (45.83)	50 (52.08)	16 (22.54)	54 (76.06)	60 (35.93)	104 (62.28)	0.01763
Gentamicin	52 (54.17)	42 (43.75)	21 (29.58)	50 (70.42)	73 (43.71)	92 (55.09)	0.001464
Cefepime	73 (76.04)	21 (21.87)	30 (42.25)	39 (54.92)	103 (61.68)	60 (35.93)	1.491e-05
Ceftazidime	70 (72.92)	23 (23.96)	29 (40.85)	40 (56.34)	99 (59.28)	63 (37.72)	2.165e-05
Aztreonam	71 (73.96)	23 (23.96)	30 (42.25)	41 (57.75)	101 (60.48)	64 (38.32)	2.237e-05
Meropenem	67 (69.79)	29 (30.21)	26 (36.62)	45 (63.38)	93 (55.69)	74 (44.31)	3.549e-05
Tetracycline	56 (58.33)	17 (17.71)	22 (28.17)	30 (42.25)	78 (46.71)	47 (28.14)	0.000151
Tigecycline	29 (30.21)	44 (45.83)	12 (16.90)	40 (56.34)	41 (24.55)	84 (50.30)	0.05599
Chloramphenicol	38 (39.58)	45 (46.88)	18 (25.35)	50 (70.42)	56 (33.53)	95 (56.89)	0.01786
Fosfomycin	66 (68.75)	26 (27.08)	12 (16.90)	42 (59.15)	78 (46.71)	68 (40.72)	6.747e-09
Nalidixic acid	74 (77.08)	22 (22.92)	33 (46.48)	38 (53.52)	107 (64.07)	60 (35.93)	7.638e-05

Based on the whole genome sequencing of all the isolates, we identified 143 types of drug resistance genes (≥80% similarity to the functionally characterized drug resistance genes), including 43 types of β-lactamase-encoding genes, such as *bla*_TEM_, *bla*_SHV_, *bla*_OXA_, *bla*_OKP_, *bla*_NDM_, *bla*_LEN_, *bla*_LAP_, *bla*_KPC_, *bla*_IMP_, *bla*_FONA_, *bla*_DHA_, *bla*_CTX-M_ and *ampC*-type genes (Supplementary Table S6). Among the β-lactamase genes, 5 types were carbapenemases, which included 102 genes: 86 *bla*_KPC-2_ (84.31%, 86/102), 8 *bla*_IMP-4_ (7.84%, 8/102), 5 *bla*_NDM-1_ (4.90%, 5/102), 2 *bla*_NDM-5_ (1.96%, 2/102), and 1 *bla*_KPC-3_ (0.98%, 1/102). Seventy-nine (91.86%, 79/86) carbapenemase-encoding genes of three types (*bla*_KPC-2_, *bla*_IMP-4_ and *bla*_KPC-3_) were present in 78 ST11 *K. pneumoniae* isolates, most of which were *bla*_KPC-2_ (96.2%, 76/79). The remaining three were *bla*_IMP-4_ (2.53%, 2/79) and *bla*_KPC-3_ (1.27%, 1/79). Apart from one ST11 *K. pneumoniae* that included both *bla*_KPC-2_ and *bla*_IMP-4_ genes, all the other 77 ST11 *K. pneumoniae* carried one carbapenem resistance gene each. The remaining *bla*_KPC-2_ genes (*n* = 10) were present in five different ST types, including five in ST23, two in ST15, and one in each of the three STs (ST86, ST107, and ST687). The remaining *bla*_IMP-4_ genes (*n* = 6) were present in six ST23 *K. pneumoniae* isolates. It is worth noting that there were five ST23 *K. pneumoniae* isolates each carrying both the *bla*_KPC-2_ and *bla*_IMP-4_ genes*. bla*_NDM-1_ was identified in two *K. pneumoniae* (ST685, ST6254) and three *K. variicola* subsp. *variicola* isolates (ST431, ST431, and ST429). *bla*_NDM-5_ was identified in two ST340 *K. pneumoniae* strains (Table 3). In addition, there were 15 different aminoglycoside-modifying enzyme genes encoding APH (6), APH (3″), APH (3″), ANT (3″), ANT (2″), AAC (6), and AAC (3′). Among the aminoglycoside resistance genes, *aadA2* was the most prevalent, and 45.51% (76/167) of the isolates carried this gene (Supplementary Table S6).

**Table 3:** The carbapenemase-encoding genes in different sequence types (STs).

Gene	Species	ST	Frequency
*bla*_KPC-2_	*K. pneumoniae*	11	76
	*K. pneumoniae*	23	5
	*K. pneumoniae*	15	2
	*K. pneumoniae*	86	1
	*K. pneumoniae*	107	1
	*K. pneumoniae*	687	1
*bla*_IMP-4_	*K. pneumoniae*	11	2
	*K. pneumoniae*	23	6
*bla*_NDM-1_	*K. pneumoniae*	685	1
	*K. pneumoniae*	6,254	1
	*K. variicola* subsp. *variicola*	431	2
	*K. variicola* subsp. *variicola*	429	1
*bla*_NDM-5_	*K. pneumoniae*	340	2
*bla*_KPC-3_	*K. pneumoniae*	11	1

### Prevalence of integrons and association between the presence of Class 1 integrons and antimicrobial susceptibility

Class 1 integrase genes were found in 57.49% (96/167) of the isolates, including 93 *K. pneumoniae* and 3 *K. variicola* subsp. *variicola*. The integron-positive isolates contained 17 ST types (15 types in *K. pneumoniae* and 2 types in *K. variicola* subsp. *variicola*), and within these *K. pneumoniae* isolates, ST11 showed the highest prevalence (75.27%, 70/93; Supplementary Table S7).

The MIC results demonstrated that the 96 isolates with class 1 integrons had a higher proportion of MDR isolates (78.13%, 75/96) than the 71 isolates with no class 1 integron (43.66%, 31/71; Table 2; Supplementary Tables S4, S5; Figure 4). The relationship between integron carriage and antimicrobial resistance levels was significant for β-lactams (cefepime, ceftazidime, aztreonam and meropenem), aminoglycosides (gentamicin and amikacin), nalidixic acid, fosfomycin, tetracycline, and chloramphenicol (*p* < 0.05). The MIC_50_ values in the integron-positive group were 2-to 512-fold higher than those in the integron-negative group. The MIC_90_ values did not show any difference between the two groups except for fosfomycin and tetracycline, which were 2-and 4-fold higher in the integron-positive group than in the integron-negative group, respectively (Table 4). In contrast, the tigecycline resistance phenotype appeared to be more frequent in the integron-negative group than in the integron-positive group.

**Table 4:** The 50 and 90% minimum inhibitory concentration (MIC_50_ and MIC_90_) values of the 11 tested antimicrobials against integron-positive and integron-negative isolates.

Antimicrobial	Integron-positive isolates (*n* = 96)		Integron-negative isolates (*n* = 71)	
	MIC_50_ (mg/L)	MIC_90_ (mg/L)	MIC_50_ (mg/L)	MIC_90_ (mg/L)
Amikacin	8	128	1	128
Gentamicin	32	128	0.25	128
Aztreonam	128	128	0.25	128
Cefepime	128	128	0.25	128
Ceftazidime	64	128	0.25	128
Chloramphenicol	16	512	8	512
Fosfomycin	256	512	64	256
Meropenem	128	128	0.25	128
Nalidixic acid	512	512	16	512
Tetracycline	16	512	8	128
Tigecycline	4	16	2	16

Furthermore, among the 143 types of drug resistance genes, 82 were present in both groups, and 36 and 25 were found only in the integron-positive and integron-negative groups, respectively (Supplementary Table S6). In the 82 types of resistance genes that appeared in both groups, the frequencies of a large number of resistance genes differed between them. The frequencies of 36 resistance genes were 1.01-to 13.68-fold higher in the integron-positive group than in the integron-negative group. These genes were mainly related to resistance to aminoglycosides [*aadA2*, *aadA5*, *aph (3′)-Ia*, *aac(3)-IId*, etc.], β-lactams (*bla*_SHV-66_, *bla*_SHV-142_, *bla*_CTX-M-65_, *bla*_CTX-M-14,_ etc.), tetracycline [*tet (A)* and *tet (D)*], fluoroquinolone (*qnrS1*, *qnrB4* and *emrB*), and so on. The prevalence of *aadA2* was higher in the integron-positive group than in the integron-negative group (75.0% vs. 5.63%). Twenty-seven types of resistance genes showed almost the same frequencies in both groups. Most (19 types) of them were efflux pump genes related to aminoglycosides (*mdtC*, *baeR*, *cpxA*, *kdpE*, etc.) and fluoroquinolone (*mdtK* and *mdtH*). The frequencies of the remaining 19 resistance genes, however, were 0.07-to 0.99-fold higher in the integron-negative group than in the integron-positive group, which included efflux pump genes related to the antimicrobials aminoglycosides (*mdtB*, *mdtA* and *crcB*) and fluoroquinolone (*emrR*), as well as β-lactams (*bla*_SHV-94_ and *bla*_SHV-33_).

The prevalence rates of 36 types of resistance genes uniquely present in the integron-positive group ranged between 1.04% (1/96) and 10.42% (10/96), and they were mainly related to the antimicrobial aminoglycosides [*aadA16*, *aac (6′)-Ib9*, *aac (6′)-Ib*, *aac (6′)-Ib10*, *ant (2″)-Ia*, etc.], fluoroquinolone (*qnrA1*, *qnrB1*, *qnrB2* and *qnrB20*), β-lactams (*bla*_SHV-11_, *bla*_SHV-27_, *bla*_IMP-4_, etc.), rifamycin (*arr-2* and *arr-3*) and so on, with the most frequent gene being *arr-3.* The prevalence of 25 types of resistance genes uniquely in the integron-negative group was between 1.41% (1/71) and 15.49% (11/71), and they were related to the antimicrobials fosfomycin and (*fosA5* and *fosA3*), fluoroquinolone (*qnrS2* and *qnrH*), β-lactams (*bla*_SHV-2_, *bla*_SHV-28_, *bla*_SHV-66_, etc.), among others, with the most frequent gene being *fosA5*.

### Analysis of gene cassettes

A total of 169 resistance gene cassettes containing 19 types of antimicrobial resistance genes were found, among which 102 resistance gene cassettes were from 79 isolates with draft genomes and 67 were from 17 isolates with complete genomes. These resistance genes were related to antimicrobials such as aminoglycosides [*aadA2*, *aadA5*, *aadA16*, *ant (2″)-Ia*, *ant (3″)-IIa, aac (6′)-Ib-cr, aac (6′)-Ib4, aac (6′)-Ib9* and *aac (6′)-Ib10*], carbapenems (*bla*_IPM-4_), β-lactams (*bla*_OXA-1_ and *bla*_OXA-10_), trimethoprim (*dfrA12*, *dfrA14*, *dfrA27*), rifampin (*arr2, arr3*), and chloramphenicol (*cmlA5* and *catB3*). The most prevalent resistance gene was *aadA2* (24.26%, 41/169), followed by *ant (3″)-IIa* (20.12%, 34/169), *aadA5* (8.88%, 15/169) and *dfrA14* (8.88%, 15/169; Table 5).

**Table 5:** Resistance gene cassettes in 96 integron-positive isolates.

Resistance gene cassette	No. of isolates
*aadA2*	41
*ant(3″)-IIa*	34
*aadA5*	15
*dfrA14*	15
*aac(6′)-Ib-cr*	11
*arr-3*	10
*aadA16*	7
*dfrA27*	7
*bla*_IMP-4_	6
*aac(6′)-Ib4*	4
*catB3*	4
*arr-2*	3
*aac(6′)-Ib9*	2
*cmlA5*	2
*dfrA12*	2
*bla*_OXA-1_	2
*bla*_OXA-10_	2
*ant(2″)-Ia*	1
*aac(6′)-Ib10*	1

To determine the structure and location of the class 1 integrons, the complete genomes of 17 integrase gene-positive isolates (KP16, KP122, KP127, KP165, KP167, KP169, KP20, KP307, KP357, KP389, KP431, KP443, KP446, KP494, KP537, KP598, and KP61) that showed a relatively wide resistance spectrum or high resistance levels or carried more resistance genes were obtained by PacBio sequencing. Among the 17 complete genomes, a total of 29 class 1 integrons with 12 different groups of gene cassette arrays were identified (Table 6; Supplementary Figure S2). Two isolates (KP169 and KP307) each contained three integrons with different structures, and eight isolates (KP16, KP122, KP127, KP167, KP357, KP389, KP431, and KP598) each harbored two integrons with different structures, whereas the remaining seven each harbored one integron. The most numerous arrays of gene cassettes were *int1*-*aadA2*-*qacEΔ1*-*sul1* (*n* = 7), *int1*-*dfrA14* (*n* = 5) and *int1-aac (6′)-Ib-cr-arr-3-dfrA27-aadA16-qacEΔ1-sul1* (*n* = 5). Analysis of the location of integrons in the 17 complete genomes revealed that except for the arrays of *int1*-*aadA2*-*qacEΔ1*-*sul1*, which were all encoded on chromosomes, the other 11 arrays were all encoded on plasmids (Table 6). Further analysis of the amino acid sequences of the integrase proteins of the 17 complete genomes showed that they were different and could be clustered into four groups with lengths of 296, 319, 337 and 370 a (Supplementary Figure S3). Each of the integrase groups contained variants of different gene cassette arrays. Interestingly, one plasmid of KP167 (pKP167-261) carried two integrons with almost the same sequences of five gene-cassette arrays (Table 6; Supplementary Figure S2).

**Table 6:** Gene cassette arrays in 17 integron-positive isolates with complete genome sequences.

Strain	Gene cassette	Size (bp)	Location	No. of isolates
KP16, KP20, KP61, KP165, KP389, KP122, KP127	*aadA2*	780	Chromosome	7
KP16, KP431, KP169, KP307^*^, KP598	*dfr14*	474	Plasmid	5
KP122, KP127, KP431, KP443, KP357	*aac(6′)-Ib-cr*-*arr-3*-*dfrA27*-*aadA16*	2,781	Plasmid	5
KP307^*^, KP357	*dfrA12*-*aadA2*	1,697	Plasmid	2
KP446, KP494	*bla*_IMP-4_-*orf*-*orf-aac(6′)-Ib9*-*catB3*	4,534	Plasmid	2
KP167	*arr2*-*cmlA5*-*bla*_OXA-10_-*ant(3″)-IIa*-*dfrA14* (duplicate)	4,454	Plasmid	1
KP389	*aadA5*	780	Plasmid	1
KP169	*ant(2″)-Ia*	648	Plasmid	1
KP598	*arr2*-*orf*-*aac(6′)-Ib-cr*	2,751	Plasmid	1
KP537	*aac(6′)-Ib-cr*-*bla*_OXA-1_-*catB3*	2,331	Plasmid	1
KP169	*aac(6′)-Ib-cr*-*bla*_OXA-1_-*catB3*-*arr-3*	2,868	Plasmid	1
KP307^*^	*aac(6′)-Ib10*-*arr-3*-*dfrA27*-*aadA16*	2,781	Plasmid	1

^*^Only the last isolate on the list (KP307) belonged to *K. variicola* subsp. *variicola*; all the others belonged *K. pneumoniae*.

### Genomic features of the KP167 plasmid pKP167-261

The complete genome of KP167 was composed of a 5.31-Mb chromosome and three plasmids. The pKP167-261 plasmid (CP098759) was 261,525 bp in size with a 51.8% GC content and harbored 280 coding sequences (CDSs). pKP167-261 was an IncFIB (K) plasmid encoding the plasmid replication genes *repFII* and *repFIB*. Based on ≥80% similarity with functionally characterized resistance genes, plasmid pKP167-261 harbored 20 resistance genes encoded in the two copies of the IS*26*-Int1 complex resistance region (one of 19,136 bp in length and the other of 19,135 bp) in the form of a tandem repeat. Each copy contained a class 1 integron region with 5 resistance genes flanked by IS*26* (AR cassette 1) [IS*26*-*int1*-*arr-2*-*cmlA5*-*bla*_OXA-10_-*ant (3″)-IIa*-*dfrA14*-IS*26* (7,340 bp in length), IS*26*-*int1*-*arr-2*-*cmlA5*-*bla*_OXA-10_-*ant (3″)-IIa*-*dfrA14*-*orf*-ΔIS*26* (7,339 bp in length)] and a fragment (11,851 bp in length) encoding five resistance genes (AR cassette 2) [*sul2*, *aph (3″)-Ib*, *aph (6)-Id*, *tet (A)* and *floR*] (Supplementary Figure S2).

**Figure 5:**
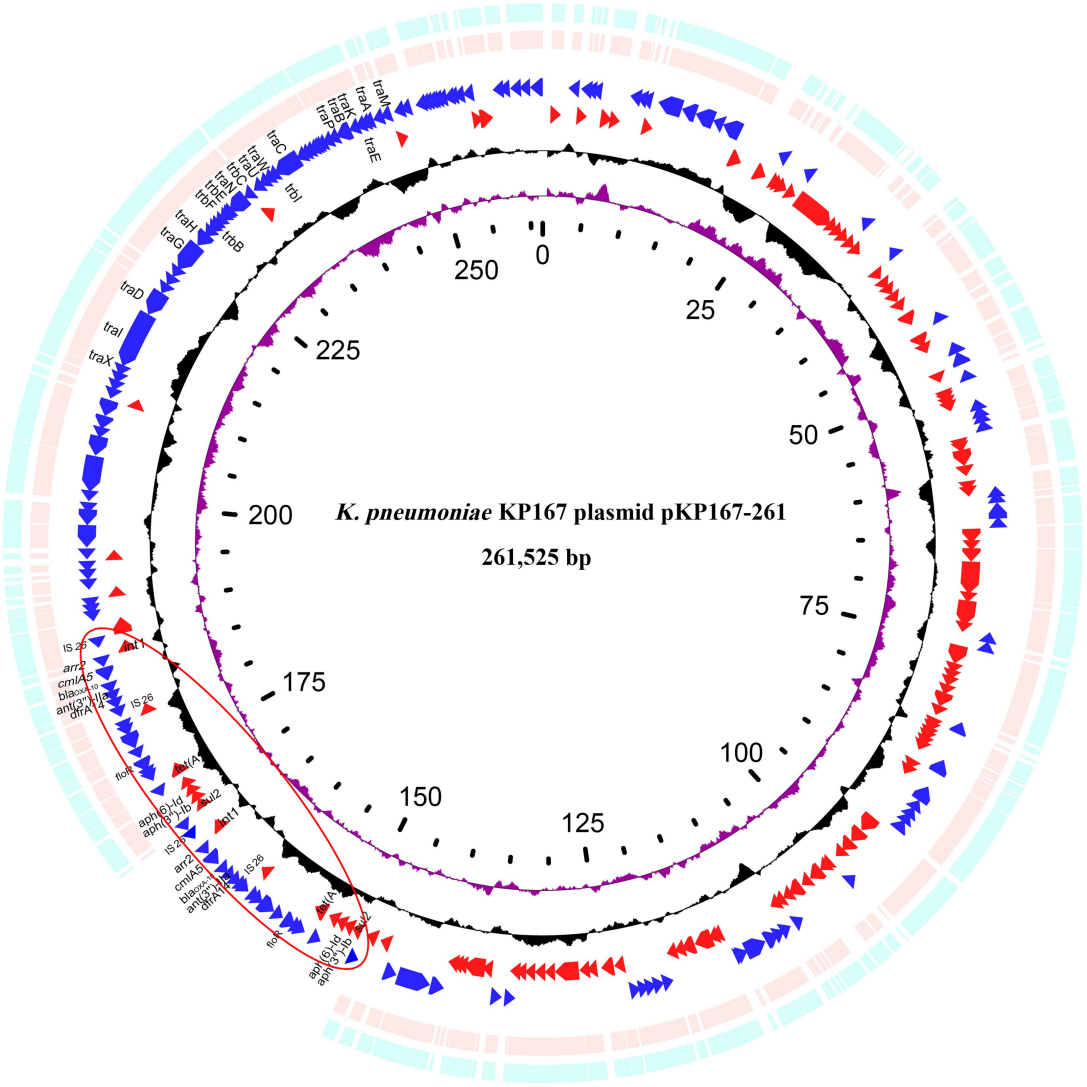
Genomic comparison of pKP167-261 with the two most similar plasmids. From outside to inside: circles 1 and 2 are homologous regions of the *K. pneumoniae* SCH6109 plasmid pSCH6109-Vir (CP050860.1) and the *K. pneumoniae* 130,411–38,618 plasmid p130411-38,618_1 (MK649826.1) compared to pKP167-261 with unmatched regions left blank; circles 3 and 4 display predicted ORFs encoded in the forward and reverse strands, respectively; circles 5 and 6 represent the GC content and GC skew, respectively; and circle 7 shows the scale in kilobases. The tandem repeat structure of IS*26*-Int1 complex resistance regions is highlighted with a red circle.

**Figure 6:**
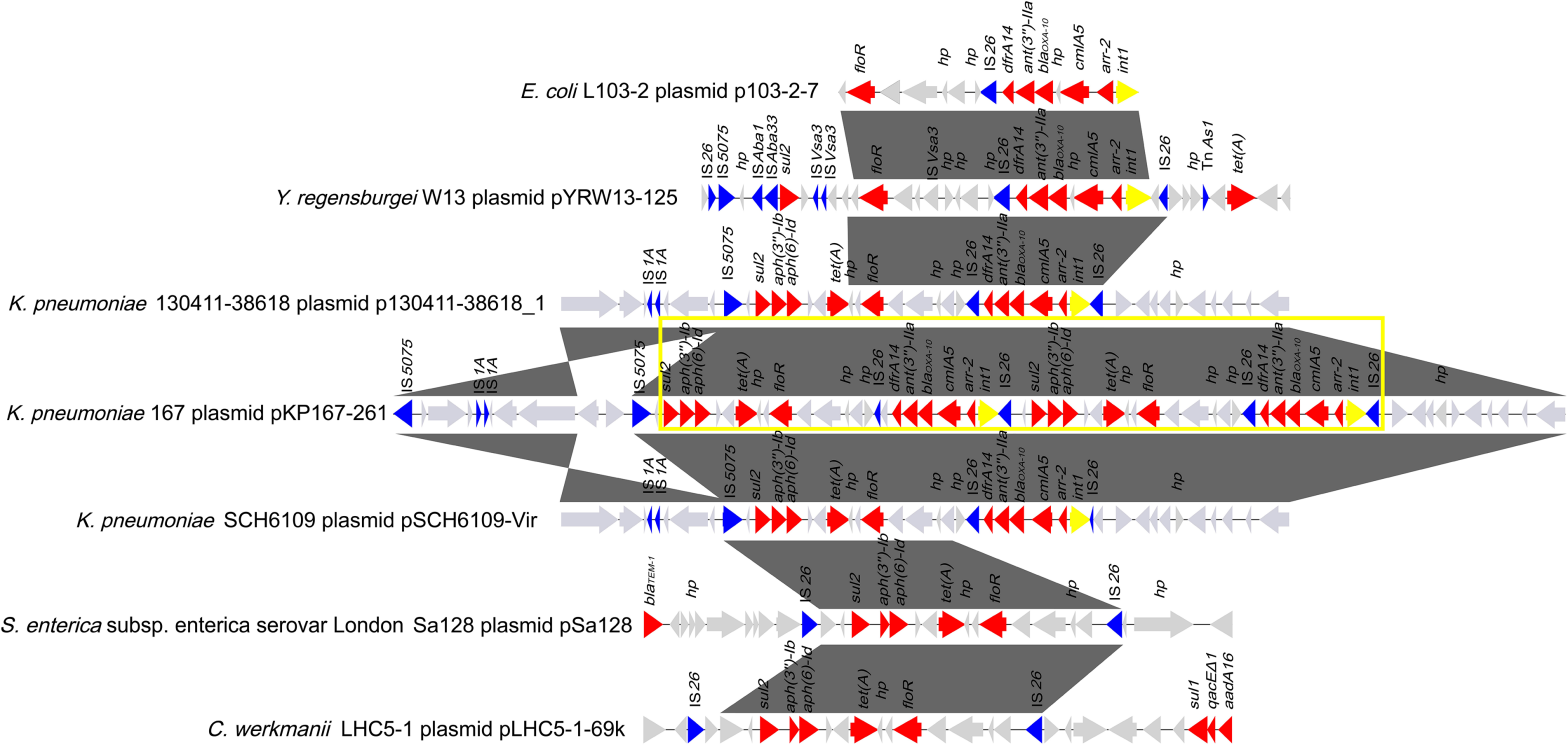
Comparative analysis of the genomic context of the two copies of the IS*26*-Int1 complex resistance region in a tandem repeat structure in pKP167-261. This structure was compared among the sequences of different sources. Genes are denoted by arrows and colored according to gene function classification. Gray shading denotes regions of homology (>90% nucleotide sequence identity). The accession numbers of the sequences are as follows: *E. coli* L103-2 plasmid p103-2-7 (CP034849.1); *Yokenella regensburgei* W13 plasmid pYRW13-125 (CP050812.1); *K. pneumoniae* 130,411–38,618 plasmid p130411-38,618_1 (MK649826.1); *K. pneumoniae* KP167 plasmid pKP167-261 (CP098759); *K. pneumoniae* SCH6109 plasmid pSCH6109-Vir (CP050860.1); *S. enterica* subsp. enterica serovar London Sa128 plasmid pSa128 (MG870194.1) and *Citrobacter werkmanii* LHC5-1 plasmid pLHC5-1-69 k (CP084291.1). The tandem repeat structure consisting of two copies of the IS*26*-Int1 complex resistance region is highlighted with a yellow box.

Eight plasmids showing relatively high nucleotide sequence similarity (coverage ≥80% and identity ≥90%) with pKP167-261 were retrieved from the NCBI nucleotide database (Table 7). All 8 plasmids originated from *K. pneumoniae*. Four of those plasmids, pCY814036-iucA (CP093152.1; 257,343 bp), p130411-38,618_1 (MK649826.1; 241,799 bp), pVir_115011 (CP089955.1; 257,157 bp) and pSCH6109-Vir (CP050860.1; 242,628 bp), shared 100% coverage and 100% identity, 100% coverage and 100% identity, 100% coverage and 100% identity and 100% coverage and 99.95% identity with pKP167-261, respectively. The main difference among pKP167-261, pCY814036-iucA, p130411-38,618_1, pSCH6109-Vir and pVir_115011 was the copy number of the IS*26*-Int1 complex resistance region mentioned above. pCY814036-iucA, pVir_115011, p130411-38,618_1 and pSCH6109-Vir each had only one copy, but pKP167-261 had two copies (Figures 5, 6). The IS*26*-Int1 complex resistance region in the plasmids pVir_115011, p130411-38,618_1 and pSCH6109-Vir were nearly identical, and they shared 100% coverage and 99.92 to 99.99% identity with that in this work (Figure 5; Supplementary Table S8).

**Table 7:** Plasmids similar to pKP167-261 in the NCBI nucleotide collection databases.

Strain	Plasmid	Size (bp)	Coverage (%)	Identity (%)	Accession No.
*Klebsiella pneumoniae* CY814036	pCY814036-iucA	257,343	100	100	CP093152.1
*Klebsiella pneumoniae* 130,411–38,618	p130411-38,618_1	241,799	100	100	MK649826.1
*Klebsiella pneumoniae* WCHKP115011	pVir_115011	257,157	100	100	CP089955.1
*Klebsiella pneumoniae* SCH6109	pSCH6109-Vir	242,628	100	99.95	CP050860.1
*Klebsiella pneumoniae* N201205880	p205880-2FIIK	229,479	84	99.97	MN824002.1
*Klebsiella pneumoniae* Kpn47	pKpn47-FIIK	248,876	86	100	MN821369.1
*Klebsiella pneumoniae* R46	pR46-270	270,566	90	100	CP035776.1
*Klebsiella pneumoniae*	pWP2-W18-ESBL-06_1 DNA	140,912	81.61	96.77	AP021930.1

### Comparative genomic analysis of IS*26*-Int1 complex resistance regions

By comparing the two copies of the class 1 integron sequences, it was found that the difference between the two was that in one of them, a nucleotide was lost in the IS*26* sequence adjacent to *dfrA14*, splitting the intact IS*26* into two parts: a small *orf* and a truncated IS*26* (ΔIS*26*; Figure 6). Using one copy of the IS*26*-Int1 complex resistance region (19,136 bp) as a query to search for homologous sequences in the NCBI nucleotide database, 112 sequences sharing ≥80% coverage and ≥ 99% identity were found. A total of 96.4% (108/112) of the sequences came from plasmids, including the nine plasmids mentioned above (Supplementary Table S8). Most of the sequences were from Enterobacteriaceae, and only a few sequences were from bacteria outside that family, such as *Aeromonas* and *Vibrio* species. No plasmid or chromosome was found to contain two copies of this IS*26*-Int1 complex resistance region.

The IS*26*-flanked class 1 integron sequences (AR cassette 1) of the IS*26*-Int1 complex resistance region identified in this work did not have 3’-CS (*qacEΔ1*/*sul1*). Analyzing the 84 sequences showing the highest similarity (coverage ≥95% and identity ≥99%) to the class 1 integron sequence [IS*26*-*int1*-*arr-2*-*cmlA5*-*bla*_OXA-10_-*ant (3″)-IIa*-*dfrA14*-IS*26*] obtained from the NCBI nucleotide database revealed that only 3 sequences were chromosomally encoded, while all the others were from plasmids. All of them came from 21 different bacterial species, among which *E. coli* was the most abundant (41.67%, 35/84), followed by *K. pneumoniae* (33.33%, 28/84). Among the 84 sequences, 13 were identical to the sequence (7,340 bp in length) identified in this work, including 7 from *E. coli*, 2 from *K. pneumoniae* and 4 from 4 different bacterial species, including *E. fergusonii, A. hydrophila*, *K. grimontii* and *S. enterica* subsp. enterica serovar Derby (Table 8). The sequence in *A. hydrophila* was encoded in the chromosome, and the other 12 were from the plasmids.

**Table 8:** Homologous sequences of antibiotic resistance (AR) cassette 1 in IS*26*-Int1 complex resistance regions.

Bacterium	Location	Coverage (%)	Identity (%)	Accession No.
*Escherichia fergusonii* HNCF11W	pHNCF11W-130 kb	100	100	CP053046.1
*Escherichia coli* H3	A	100	100	CP010168.1
*Klebsiella pneumoniae* CY814036	pCY814036-iucA	100	100	CP093152.1
*Klebsiella grimontii* 2,481,359	p2481359-2	100	100	CP067382.1
*Escherichia coli* LD67-1	pLD67-1-157 kb	100	100	CP061187.1
*Escherichia coli* 98.1	p1	100	100	CP059954.1
*Escherichia coli*	pIncX1_p1	100	100	MN783746.1
*Escherichia coli* L103-2	p103-2-7	100	100	CP034849.1
*Escherichia coli* CT29	p. CT29-P4	100	100	CP032077.1
*Escherichia coli* RCAD0514	pRCAD0514EC-1	100	100	CP034107.1
*Klebsiella pneumoniae* fekpn2511	pfekpn2511-3	100	100	CP068975.1
*Salmonella enterica* subsp. enterica serovar Derby SA1982	Unnamed plasmid	100	100	MT513102.1
*Aeromonas hydrophila* ZYAH75	Chromosome	100	100	CP016990.1

When searching for homologous sequences of the five-resistance gene encoding fragments (AR cassette 2; 11,851 bp) of the IS*26*-Int1 complex resistance region, a total of 361 sequences sharing high nucleotide sequence similarity (coverage ≥95% and identity ≥99%) were obtained from the NCBI nucleotide database. All the sequences were encoded on plasmids except nine, which were on chromosomes. They were from 80 different species, with the greatest number derived from *E. coli* (32.69%, 118/361), followed by *K. pneumoniae* (13.85%, 50/361). Four sequences (MK649826.1, CP093152.1, CP089955.1, CP068973.1) were identical to those in this work, and all of them were from plasmids of *K. pneumoniae* (Table 9).

**Table 9:** Homologous sequences of antibiotic resistance (AR) cassette 2 of IS26-Int1 complex resistance regions.

Bacterium	Location	Coverage (%)	Identity (%)	Accession No.
*Klebsiella pneumoniae* 130,411–38,618	p130411-38,618_1	100	100	MK649826.1
*Klebsiella pneumoniae* CY814036	pCY814036-iucA,	100	100	CP093152.1
*Klebsiella pneumoniae* WCHKP115011	pVir_115011	100	100	CP089955.1
*Klebsiella pneumoniae* fekpn2511	pfekpn2511-1	100	100	CP068973.1

## Discussion

In this study, based on ANI analysis, 167 clinically identified *K. pneumoniae* isolates were classified as four different *Klebsiella* species, including three species of the *Klebsiella pneumoniae* complex group (*K. pneumoniae*, *K. variicola* subsp. *variicola* and *K. quasipneumoniae* subsp. *similipneumoniae*) and *K. aerogenes*. *K. variicola* and *K. quasipneumoniae* are relatively common pathogens causing hospital-acquired infections, but traditional clinical laboratory methods (MALDI-TOF MS, multilocus sequence typing, or capsule genotyping) may misclassify them as *K. pneumoniae*, which would underestimate the clinical infection they cause and the actual prevalence (Wyres et al., 2020; Ohama et al., 2022).

Forty-one STs, including five novel STs, were identified in 152 *K. pneumoniae* isolates, of which ST11 (51.32%, 78/152) was identified as the dominant sequence type in the hospital, which is consistent with previous results (Kong et al., 2020). In the present study, most ST11 *K. pneumoniae* strains carried class 1 integrons (89.74%, 70/78) and were MDR (70.51%, 55/78). Additionally, more than 90% of the carbapenemase genes (mainly *bla*_KPC-2_) were encoded in ST11 *K. pneumoniae*. From the phylogenetic analysis (Figure 3), clonal outbreaks of ST11 *K. pneumoniae* could be found in some departments, especially in the Intensive Care Unit (A) and Hematology Department (B). The clonal transmission events may result from more complex pathways, intermediate patients, or environmental sources; these factors need further study (Sui et al., 2018). As an important opportunistic pathogen associated with nosocomial bacterial infections, studies have revealed that ST11 *K. pneumoniae* has a high prevalence of virulence factors favoring binding, biofilm formation, colonization and escape from phagocytosis, which can allow clones of this pathogen to successfully spread worldwide (Andrade et al., 2014; Liao et al., 2020b). During the clonal spread of ST11 *K. pneumoniae* strains, the diverse genomic structures of clinical pathogens may help to adapt to the complex and strong selective pressure of the clinical environment (Barrios-Camacho et al., 2019; Rodriguez-Medina et al., 2020).

More than half (57.49%) of the clinical *Klebsiella* isolates from Zhejiang, China, carried class 1 integrons, which was slightly higher than those previously reported in other districts, such as Beijing, or elsewhere in China (Li et al., 2013; Liao et al., 2020a). Compared with the class 1 integron–negative group, the class 1 integron-positive group exhibited much higher resistance rates against a number of antimicrobials, such as nalidixic acid (77.08% vs. 46.48%), cefepime (76.04% vs. 42.25%), aztreonam (73.96% vs. 42.25%) and ceftazidime (72.92% vs. 40.85%), and likewise, the proportion of MDR isolates was significantly higher in the class 1 integron-positive group (78.12% vs. 46.48%). In addition, more types and numbers of resistance genes were identified in class 1 integron-positive isolates than in class 1 integron-negative isolates, such as *aadA2* (75% vs. 5.65%), *bla*_KPC-2_ (77.08% vs. 16.90%), and *bla*_SHV-1_ (73.96% vs. 12.68%), which made the class 1 integron-positive group show higher MIC levels for the corresponding antimicrobials. Similar to transposons, class 1 integrons can capture resistance genes from bacteria of various sources (Fluit and Schmitz, 2004; Firoozeh et al., 2019; Farhadi et al., 2021). It plays an important role in resistance gene spreading by means of horizontal gene transfer between bacteria of different species or genera, resulting in the increasing emergence of MDR bacteria, especially clinical pathogens (Fluit and Schmitz, 2004; Firoozeh et al., 2019; Farhadi et al., 2021).

Twelve groups of 29 complete class 1 integrons were identified in 17 isolates with complete genome sequences. Almost all class 1 integrons or resistance gene cassettes from the remaining class 1 integrase gene-positive strains (without complete genome sequences) could be mapped to one of the 12 groups of class 1 integrons. These 12 groups of integrons were all present in *Enterobacteriaceae*, especially *E. coli* and *K. pneumoniae* (Li et al., 2013; Liu et al., 2015). The class 1 integron *int1*-*aadA2*-*qacEΔ1*-*sul1* was found to be encoded in the chromosomes of seven *K. pneumoniae* of two ST types, ST11 and ST340. NCBI nucleotide database searching revealed that this integron was also found in 108 *K. pneumoniae* chromosomes and two plasmids, one of which came from *K. pneumoniae*, while the other came from *Enterobacter asburiae* (Supplementary Table S9). This suggested that this integron had undergone horizontal transfer between different bacterial species.

The integron carrying the carbapenemase gene *bla*_IMP-4_ (*bla*_IMP-4_-*orf*-*orf-aac (6′)-Ib9*-*catB3*) was found in two *K. pneumoniae* isolates (KP446 and KP494). A similar integron was found in the plasmids of 9 *Klebsiella* strains and 1 *E. asburiae* strain available in public nucleotide databases (Supplementary Table S10). The carbapenem resistance gene carried by the integrons and encoded on the plasmids may lead to a broadened distribution of carbapenem resistance within and between species of different genera and may increase the severity of problems caused by MDR bacteria (van Duin and Doi, 2017).

Interestingly, one plasmid contained two copies of the IS*26*-Int1 complex resistance region, with each IS*26*-Int1 complex resistance region consisting of a class 1 integron fragment (AR cassette 1) and a multidrug resistance fragment (AR cassette 2). Previously, IS*26* has been involved in the amplification of resistance gene-related sequences (Hansen et al., 2019; Harmer et al., 2022). In the present study, comparative genomic analysis revealed that the single IS*26*-Int1 complex resistance region was found in a variety of bacterial genera in the family *Enterobacteriaceae* (*Escherichia*, *Klebsiella*, *Salmonella*) and was mostly encoded on plasmids, but none of them contained this double copy structure (Garza-Ramos et al., 2009; Ji et al., 2022). The BLASTN search of AR cassette 1 and AR cassette 2 suggested that both structures are widely present in different bacterial species and associated with IS*26*. In the present study, we did not identify this duplication in another genome, perhaps because of a high fitness cost or because strains carrying this structure have not been submitted (Adler et al., 2014; McGann et al., 2014). Additional work is necessary to determine the implications of this duplication structure.

Gene duplication/amplification constitutes an important adaptive mechanism in bacteria, and under the strength of clinal antibiotic selection pressure, amplification of antibiotic resistance genes could have a specific clinical impact, including leading to higher expression of these genes and levels of resistance to antibiotics, ultimately contributing to bacterial survival (Sandegren and Andersson, 2009). Bacteria with multiple copies of identical resistance genes or gene arrays have been frequently identified. The recombinant with four copies of *bla*_GES-5_ had a 2-to 4-fold increase in its MIC levels for the tested β-lactam antimicrobials compared with that carrying one copy of *bla*_GES-5_, and *bla*_GES-5_ was expressed more abundantly in the former (by approximately twofold) than in the latter. The presence of multiple copies of the *bla*_OXA-58_ gene resulted in high-level resistance to carbapenems in *Acinetobacter baumannii*, and duplication of a 36.4 kb region encompassing *bla*_SHV-11_ in a clinical isolate of *K. pneumoniae* increased a 16-fold MIC level to amoxicillin (Bertini et al., 2007; Duvernay et al., 2011; Xu et al., 2018). Therefore, it is essential to identify and monitor the occurrence of resistance gene duplication/amplification and its possible impact on the MIC and treatment failure of relevant antibiotics.

## Conclusion

In this study, based on whole genome sequencing, the species identification of 167 clinical *Klebsiella* isolates revealed three additional species: *K. variicola* subsp. *variicola*, *K. quasipneumoniae* subsp. *similipneumoniae* and *K. aerogenes* identified by ANI that were not identified by either the common clinical laboratory method or 16S rRNA gene homology analysis. Accurate identification of *Klebsiella* species contributed to the clinical monitoring of the prevalence of pathogenic bacteria and the designation and implementation of novel control strategies. A total of 169 resistance gene cassettes encoding 19 types of resistance genes were found in 96 integrase gene-positive isolates. Among the 17 complete genomes, 12 groups of class 1 integrons were identified, among which one group was encoded on chromosomes, while the others were encoded on the plasmids. One plasmid carrying two copies of the IS*26*-Int1 complex resistance region in a tandem repeat form is reported for the first time in this work. These findings indicate that continuing research on the genome structures of pathogenic bacteria, focusing on resistance-related sequence structures, is of great importance for elucidating the molecular backgrounds of pathogens and the mechanisms through which resistance emerges and spreads.

## Data availability statement

The datasets presented in this study can be found in online repositories. The names of the repository/repositories and accession number (s) can be found in the article/Supplementary material.

## Ethics statement

Individual patient data were not involved, and only anonymous residual clinical samples obtained during routine hospital laboratory procedures were used in this study. This study was approved by the ethics committee of Zhejiang Hospital, Hangzhou, Zhejiang, China.

## Author contributions

QB, YH, TX, and JlL conceived and designed the experiments. LaW, MZ, CY, YZ, XH, LiW, JX, and JwL performed the experiments. LaW, MZ, and JlL performed data analysis and interpretation. LaW, MZ, QB, TX, and JlL drafted the manuscript. All authors contributed to the article and approved the submitted version.

## Funding

This study was supported by the Zhejiang Provincial Natural Science Foundation of China (LGF19H200003); the Science & Technology Project of Wenzhou City, China (N20210001, Y2020112); the Natural Science Foundation of China (81960381); and the Science and Technology Planning Project of Zhejiang Province (LGN19C180002).

## Conflict of interest

The authors declare that the research was conducted in the absence of any commercial or financial relationships that could be construed as a potential conflict of interest.

## Publisher’s note

All claims expressed in this article are solely those of the authors and do not necessarily represent those of their affiliated organizations, or those of the publisher, the editors and the reviewers. Any product that may be evaluated in this article, or claim that may be made by its manufacturer, is not guaranteed or endorsed by the publisher.

## Footnotes

^1^https://www.fda.gov/drugs/development-resources/antibacterial-susceptibility-test-interpretive-criteria

^2^https://server.Gview.ca/
