# Supplementary material for: Class 1 integrons and multiple mobile genetic elements in clinical isolates of the Klebsiella pneumoniae complex from a tertiary hospital in eastern China
Source: Front Microbiol. 2023 Mar 6;14:985102. doi: 10.3389/fmicb.2023.985102 (PMC10026359; doi:10.3389/fmicb.2023.985102)
Supplement: Supplementary file 1 [file Data_Sheet_1.zip › Supplemental Dataset.pdf]

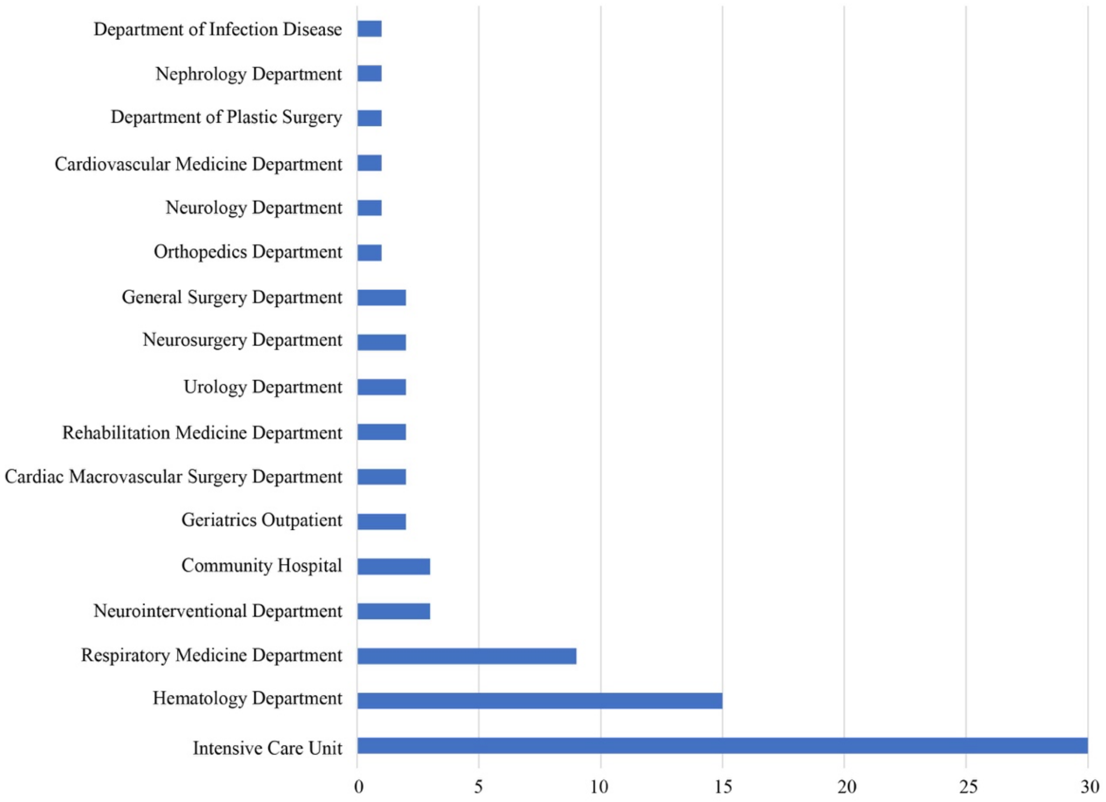

Figure S1. Information on the departments where ST11 *Klebsiella* isolates were found.

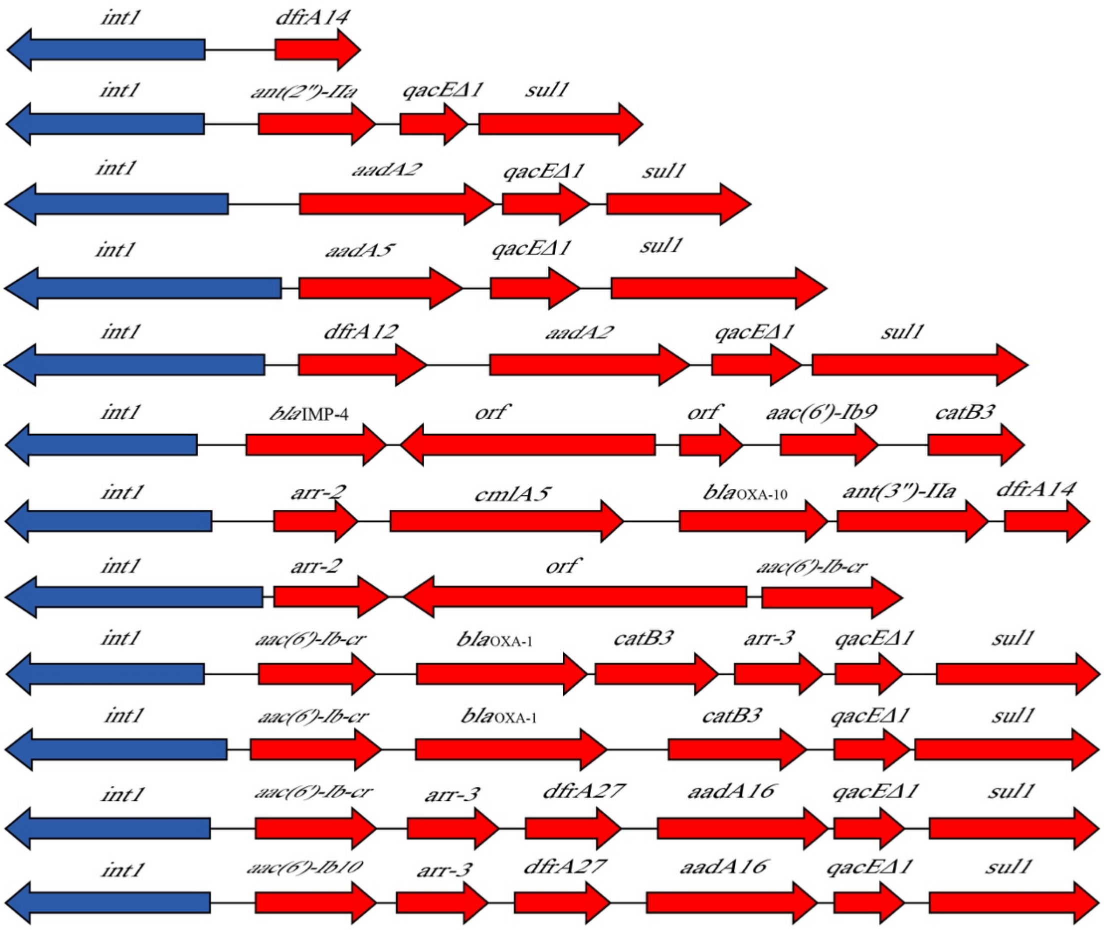

Figure S2. Representative gene cassette arrays of 12 groups of integrons from the complete genomes of 17 *Klebsiella* isolates.

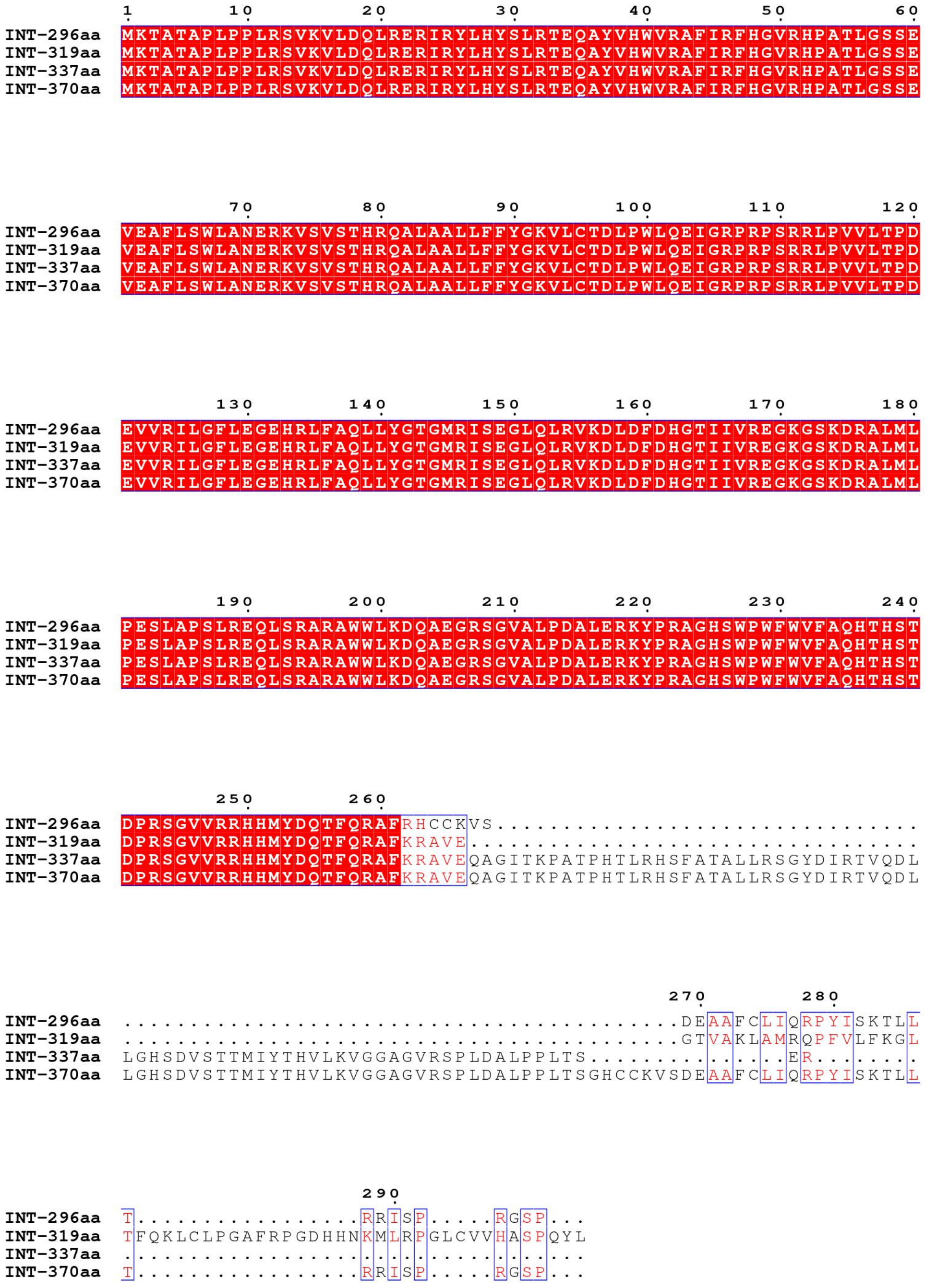

Figure S3. Sequence comparison assay of integrases.

Table S1. Results of 16S rRNA gene homology analysis of 167 *Klebsiella* isolates.

Strain	Length (bp)	Reference sequences (Accession No.)	Coverage (%)	Identity (%)	Similarity (%)
KP472	1536	*Klebsiella pneumoniae* strain DSM 30104 16S ribosomal RNA, partial sequence (OM403526.1)	99.54	99.87	99.41
KP214	1536	*Klebsiella pneumoniae* strain DSM 30104 16S ribosomal RNA, partial sequence (OM403526.1)	99.54	99.8	99.34
KP233	1536	*Klebsiella pneumoniae* strain DSM 30104 16S ribosomal RNA, partial sequence (OM403526.1)	99.54	99.8	99.34
KP297	1536	*Klebsiella pneumoniae* strain DSM 30104 16S ribosomal RNA, partial sequence (OM403526.1)	99.54	99.8	99.34
KP382	1536	*Klebsiella pneumoniae* strain DSM 30104 16S ribosomal RNA, partial sequence (OM403526.1)	99.54	99.8	99.34
KP399	1536	*Klebsiella pneumoniae* strain DSM 30104 16S ribosomal RNA, partial sequence (OM403526.1)	99.54	99.8	99.34
KP410	1536	*Klebsiella pneumoniae* strain DSM 30104 16S ribosomal RNA, partial sequence (OM403526.1)	99.54	99.8	99.34
KP450	1536	*Klebsiella pneumoniae* strain DSM 30104 16S ribosomal RNA, partial sequence (OM403526.1)	99.54	99.8	99.34
KP567	1536	*Klebsiella pneumoniae* strain DSM 30104 16S ribosomal RNA, partial sequence (OM403526.1)	99.54	99.8	99.34
KP588	1536	*Klebsiella pneumoniae* strain DSM 30104 16S ribosomal RNA, partial sequence (OM403526.1)	99.54	99.8	99.34

KP561	1063	*Klebsiella pneumoniae* strain DSM 30104 16S ribosomal RNA, partial sequence (OM403526.1)	100	99.77	99.77
KP565	1063	*Klebsiella pneumoniae* strain DSM 30104 16S ribosomal RNA, partial sequence (OM403526.1)	100	99.77	99.77
KP220	1536	*Klebsiella pneumoniae* strain DSM 30104 16S ribosomal RNA, partial sequence (OM403526.1)	99.8	99.74	99.54
KP363	1536	*Klebsiella pneumoniae* strain DSM 30104 16S ribosomal RNA, partial sequence (OM403526.1)	99.8	99.74	99.54
KP437	1536	*Klebsiella pneumoniae* strain DSM 30104 16S ribosomal RNA, partial sequence (OM403526.1)	99.54	99.74	99.28
KP514	1536	*Klebsiella pneumoniae* strain DSM 30104 16S ribosomal RNA, partial sequence (OM403526.1)	99.8	99.74	99.54
KP585	1536	*Klebsiella pneumoniae* strain DSM 30104 16S ribosomal RNA, partial sequence (OM403526.1)	99.54	99.74	99.28
KP586	1536	*Klebsiella pneumoniae* strain DSM 30104 16S ribosomal RNA, partial sequence (OM403526.1)	99.54	99.74	99.28
KP94	1536	*Klebsiella pneumoniae* strain DSM 30104 16S ribosomal RNA, partial sequence (OM403526.1)	99.54	99.74	99.28
KP115	1063	*Klebsiella pneumoniae* strain DSM 30104 16S ribosomal RNA, partial sequence (OM403526.1)	99.34	99.72	99.06
KP227	1063	*Klebsiella pneumoniae* strain DSM 30104 16S ribosomal RNA, partial sequence (OM403526.1)	99.34	99.72	99.06

KP246	1063	*Klebsiella pneumoniae* strain DSM 30104 16S ribosomal RNA, partial sequence (OM403526.1)	99.34	99.72	99.06
KP276	1063	*Klebsiella pneumoniae* strain DSM 30104 16S ribosomal RNA, partial sequence (OM403526.1)	99.34	99.72	99.06
KP452	1063	*Klebsiella pneumoniae* strain DSM 30104 16S ribosomal RNA, partial sequence (OM403526.1)	99.34	99.72	99.06
KP50	1063	*Klebsiella pneumoniae* strain DSM 30104 16S ribosomal RNA, partial sequence (OM403526.1)	99.34	99.72	99.06
KP253	1536	*Klebsiella pneumoniae* strain DSM 30104 16S ribosomal RNA, partial sequence (OM403526.1)	99.54	99.67	99.21
KP259	1536	*Klebsiella pneumoniae* strain DSM 30104 16S ribosomal RNA, partial sequence (OM403526.1)	99.8	99.67	99.47
KP284	1536	*Klebsiella pneumoniae* strain DSM 30104 16S ribosomal RNA, partial sequence (OM403526.1)	99.8	99.67	99.47
KP298	1536	*Klebsiella pneumoniae* strain DSM 30104 16S ribosomal RNA, partial sequence (OM403526.1)	99.54	99.67	99.21
KP329	1536	*Klebsiella pneumoniae* strain DSM 30104 16S ribosomal RNA, partial sequence (OM403526.1)	99.54	99.67	99.21
KP352	1536	*Klebsiella pneumoniae* strain DSM 30104 16S ribosomal RNA, partial sequence (OM403526.1)	99.54	99.67	99.21
KP356	1536	*Klebsiella pneumoniae* strain DSM 30104 16S ribosomal RNA, partial sequence (OM403526.1)	99.54	99.67	99.21

KP357	1536	*Klebsiella pneumoniae* strain DSM 30104 16S ribosomal RNA, partial sequence (OM403526.1)	99.8	99.67	99.47
KP365	1536	*Klebsiella pneumoniae* strain DSM 30104 16S ribosomal RNA, partial sequence (OM403526.1)	99.54	99.67	99.21
KP393	1536	*Klebsiella pneumoniae* strain DSM 30104 16S ribosomal RNA, partial sequence (OM403526.1)	99.54	99.67	99.21
KP483	1536	*Klebsiella pneumoniae* strain DSM 30104 16S ribosomal RNA, partial sequence (OM403526.1)	99.54	99.67	99.21
KP494	1536	*Klebsiella pneumoniae* strain DSM 30104 16S ribosomal RNA, partial sequence (OM403526.1)	99.54	99.67	99.21
KP592	1536	*Klebsiella pneumoniae* strain DSM 30104 16S ribosomal RNA, partial sequence (OM403526.1)	99.54	99.67	99.21
KP598	1536	*Klebsiella pneumoniae* strain DSM 30104 16S ribosomal RNA, partial sequence (OM403526.1)	99.54	99.67	99.21
KP307	1063	*Klebsiella pneumoniae* strain DSM 30104 16S ribosomal RNA, partial sequence (OM403526.1)	99.34	99.62	98.96
KP538	1063	*Klebsiella pneumoniae* strain DSM 30104 16S ribosomal RNA, partial sequence (OM403526.1)	99.34	99.62	98.96
KP553	1063	*Klebsiella pneumoniae* strain DSM 30104 16S ribosomal RNA, partial sequence (OM403526.1)	99.34	99.62	98.96
KP112	1536	*Klebsiella pneumoniae* strain DSM 30104 16S ribosomal RNA, partial sequence (OM403526.1)	99.54	99.61	99.15

KP117	1536	*Klebsiella pneumoniae* strain DSM 30104 16S ribosomal RNA, partial sequence (OM403526.1)	99.54	99.61	99.15
KP120	1536	*Klebsiella pneumoniae* strain DSM 30104 16S ribosomal RNA, partial sequence (OM403526.1)	99.54	99.61	99.15
KP149	1536	*Klebsiella pneumoniae* strain DSM 30104 16S ribosomal RNA, partial sequence (OM403526.1)	99.54	99.61	99.15
KP153	1536	*Klebsiella pneumoniae* strain DSM 30104 16S ribosomal RNA, partial sequence (OM403526.1)	99.54	99.61	99.15
KP159	1536	*Klebsiella pneumoniae* strain DSM 30104 16S ribosomal RNA, partial sequence (OM403526.1)	99.54	99.61	99.15
KP16	1536	*Klebsiella pneumoniae* strain DSM 30104 16S ribosomal RNA, partial sequence (OM403526.1)	99.54	99.61	99.15
KP166	1536	*Klebsiella pneumoniae* strain DSM 30104 16S ribosomal RNA, partial sequence (OM403526.1)	99.54	99.61	99.15
KP169	1536	*Klebsiella pneumoniae* strain DSM 30104 16S ribosomal RNA, partial sequence (OM403526.1)	99.54	99.61	99.15
KP181	1536	*Klebsiella pneumoniae* strain DSM 30104 16S ribosomal RNA, partial sequence (OM403526.1)	99.54	99.61	99.15
KP186	1536	*Klebsiella pneumoniae* strain DSM 30104 16S ribosomal RNA, partial sequence (OM403526.1)	99.54	99.61	99.15
KP20	1536	*Klebsiella pneumoniae* strain DSM 30104 16S ribosomal RNA, partial sequence (OM403526.1)	99.54	99.61	99.15

KP202	1536	*Klebsiella pneumoniae* strain DSM 30104 16S ribosomal RNA, partial sequence (OM403526.1)	99.54	99.61	99.15
KP208	1536	*Klebsiella pneumoniae* strain DSM 30104 16S ribosomal RNA, partial sequence (OM403526.1)	99.54	99.61	99.15
KP226	1536	*Klebsiella pneumoniae* strain DSM 30104 16S ribosomal RNA, partial sequence (OM403526.1)	99.54	99.61	99.15
KP241	1536	*Klebsiella pneumoniae* strain DSM 30104 16S ribosomal RNA, partial sequence (OM403526.1)	99.54	99.61	99.15
KP255	1536	*Klebsiella pneumoniae* strain DSM 30104 16S ribosomal RNA, partial sequence (OM403526.1)	99.54	99.61	99.15
KP256	1536	*Klebsiella pneumoniae* strain DSM 30104 16S ribosomal RNA, partial sequence (OM403526.1)	99.54	99.61	99.15
KP260	1536	*Klebsiella pneumoniae* strain DSM 30104 16S ribosomal RNA, partial sequence (OM403526.1)	99.54	99.61	99.15
KP261	1536	*Klebsiella pneumoniae* strain DSM 30104 16S ribosomal RNA, partial sequence (OM403526.1)	99.54	99.61	99.15
KP266	1536	*Klebsiella pneumoniae* strain DSM 30104 16S ribosomal RNA, partial sequence (OM403526.1)	99.54	99.61	99.15
KP270	1536	*Klebsiella pneumoniae* strain DSM 30104 16S ribosomal RNA, partial sequence (OM403526.1)	99.8	99.61	99.41
KP279	1536	*Klebsiella pneumoniae* strain DSM 30104 16S ribosomal RNA, partial sequence (OM403526.1)	99.54	99.61	99.15

KP281	1536	*Klebsiella pneumoniae* strain DSM 30104 16S ribosomal RNA, partial sequence (OM403526.1)	99.54	99.61	99.15
KP283	1536	*Klebsiella pneumoniae* strain DSM 30104 16S ribosomal RNA, partial sequence (OM403526.1)	99.54	99.61	99.15
KP288	1536	*Klebsiella pneumoniae* strain DSM 30104 16S ribosomal RNA, partial sequence (OM403526.1)	99.54	99.61	99.15
KP301	1536	*Klebsiella pneumoniae* strain DSM 30104 16S ribosomal RNA, partial sequence (OM403526.1)	99.54	99.61	99.15
KP302	1536	*Klebsiella pneumoniae* strain DSM 30104 16S ribosomal RNA, partial sequence (OM403526.1)	99.54	99.61	99.15
KP315	1536	*Klebsiella pneumoniae* strain DSM 30104 16S ribosomal RNA, partial sequence (OM403526.1)	99.54	99.61	99.15
KP332	1536	*Klebsiella pneumoniae* strain DSM 30104 16S ribosomal RNA, partial sequence (OM403526.1)	99.54	99.61	99.15
KP340	1536	*Klebsiella pneumoniae* strain DSM 30104 16S ribosomal RNA, partial sequence (OM403526.1)	99.54	99.61	99.15
KP354	1536	*Klebsiella pneumoniae* strain DSM 30104 16S ribosomal RNA, partial sequence (OM403526.1)	99.54	99.61	99.15
KP359	1536	*Klebsiella pneumoniae* strain DSM 30104 16S ribosomal RNA, partial sequence (OM403526.1)	99.54	99.61	99.15
KP36	1536	*Klebsiella pneumoniae* strain DSM 30104 16S ribosomal RNA, partial sequence (OM403526.1)	99.8	99.61	99.41

KP360	1536	*Klebsiella pneumoniae* strain DSM 30104 16S ribosomal RNA, partial sequence (OM403526.1)	99.54	99.61	99.15
KP377	1536	*Klebsiella pneumoniae* strain DSM 30104 16S ribosomal RNA, partial sequence (OM403526.1)	99.54	99.61	99.15
KP384	1536	*Klebsiella pneumoniae* strain DSM 30104 16S ribosomal RNA, partial sequence (OM403526.1)	99.8	99.61	99.41
KP385	1536	*Klebsiella pneumoniae* strain DSM 30104 16S ribosomal RNA, partial sequence (OM403526.1)	99.54	99.61	99.15
KP387	1536	*Klebsiella pneumoniae* strain DSM 30104 16S ribosomal RNA, partial sequence (OM403526.1)	99.8	99.61	99.41
KP388	1536	*Klebsiella pneumoniae* strain DSM 30104 16S ribosomal RNA, partial sequence (OM403526.1)	99.54	99.61	99.15
KP394	1536	*Klebsiella pneumoniae* strain DSM 30104 16S ribosomal RNA, partial sequence (OM403526.1)	99.54	99.61	99.15
KP404	1536	*Klebsiella pneumoniae* strain DSM 30104 16S ribosomal RNA, partial sequence (OM403526.1)	99.54	99.61	99.15
KP408	1536	*Klebsiella pneumoniae* strain DSM 30104 16S ribosomal RNA, partial sequence (OM403526.1)	99.54	99.61	99.15
KP423	1536	*Klebsiella pneumoniae* strain DSM 30104 16S ribosomal RNA, partial sequence (OM403526.1)	99.54	99.61	99.15
KP430	1536	*Klebsiella pneumoniae* strain DSM 30104 16S ribosomal RNA, partial sequence (OM403526.1)	99.54	99.61	99.15

KP431	1536	*Klebsiella pneumoniae* strain DSM 30104 16S ribosomal RNA, partial sequence (OM403526.1)	99.54	99.61	99.15
KP432	1536	*Klebsiella pneumoniae* strain DSM 30104 16S ribosomal RNA, partial sequence (OM403526.1)	99.54	99.61	99.15
KP459	1536	*Klebsiella pneumoniae* strain DSM 30104 16S ribosomal RNA, partial sequence (OM403526.1)	99.54	99.61	99.15
KP461	1536	*Klebsiella pneumoniae* strain DSM 30104 16S ribosomal RNA, partial sequence (OM403526.1)	99.54	99.61	99.15
KP470	1536	*Klebsiella pneumoniae* strain DSM 30104 16S ribosomal RNA, partial sequence (OM403526.1)	99.54	99.61	99.15
KP495	1536	*Klebsiella pneumoniae* strain DSM 30104 16S ribosomal RNA, partial sequence (OM403526.1)	99.54	99.61	99.15
KP537	1536	*Klebsiella pneumoniae* strain DSM 30104 16S ribosomal RNA, partial sequence (OM403526.1)	99.54	99.61	99.15
KP569	1536	*Klebsiella pneumoniae* strain DSM 30104 16S ribosomal RNA, partial sequence (OM403526.1)	99.8	99.61	99.41
KP576	1536	*Klebsiella pneumoniae* strain DSM 30104 16S ribosomal RNA, partial sequence (OM403526.1)	99.8	99.61	99.41
KP579	1536	*Klebsiella pneumoniae* strain DSM 30104 16S ribosomal RNA, partial sequence (OM403526.1)	99.54	99.61	99.15
KP580	1536	*Klebsiella pneumoniae* strain DSM 30104 16S ribosomal RNA, partial sequence (OM403526.1)	99.8	99.61	99.41

KP583	1536	*Klebsiella pneumoniae* strain DSM 30104 16S ribosomal RNA, partial sequence (OM403526.1)	99.8	99.61	99.41
KP593	1536	*Klebsiella pneumoniae* strain DSM 30104 16S ribosomal RNA, partial sequence (OM403526.1)	99.54	99.61	99.15
KP61	1536	*Klebsiella pneumoniae* strain DSM 30104 16S ribosomal RNA, partial sequence (OM403526.1)	99.54	99.61	99.15
KP67	1536	*Klebsiella pneumoniae* strain DSM 30104 16S ribosomal RNA, partial sequence (OM403526.1)	99.54	99.61	99.15
KP80	1536	*Klebsiella pneumoniae* strain DSM 30104 16S ribosomal RNA, partial sequence (OM403526.1)	99.54	99.61	99.15
KP9	1536	*Klebsiella pneumoniae* strain DSM 30104 16S ribosomal RNA, partial sequence (OM403526.1)	99.54	99.61	99.15
KP113	1536	*Klebsiella pneumoniae* strain DSM 30104 16S ribosomal RNA, partial sequence (OM403526.1)	99.8	99.54	99.34
KP141	1536	*Klebsiella pneumoniae* strain DSM 30104 16S ribosomal RNA, partial sequence (OM403526.1)	99.8	99.54	99.34
KP231	1536	*Klebsiella pneumoniae* strain DSM 30104 16S ribosomal RNA, partial sequence (OM403526.1)	99.8	99.54	99.34
KP325	1536	*Klebsiella pneumoniae* strain DSM 30104 16S ribosomal RNA, partial sequence (OM403526.1)	99.8	99.54	99.34
KP345	1536	*Klebsiella pneumoniae* strain DSM 30104 16S ribosomal RNA, partial sequence (OM403526.1)	99.54	99.54	99.08

KP389	1536	*Klebsiella pneumoniae* strain DSM 30104 16S ribosomal RNA, partial sequence (OM403526.1)	99.8	99.54	99.34
KP443	1536	*Klebsiella pneumoniae* strain DSM 30104 16S ribosomal RNA, partial sequence (OM403526.1)	99.54	99.54	99.08
KP445	1536	*Klebsiella pneumoniae* strain DSM 30104 16S ribosomal RNA, partial sequence (OM403526.1)	99.8	99.54	99.34
KP446	1536	*Klebsiella pneumoniae* strain DSM 30104 16S ribosomal RNA, partial sequence (OM403526.1)	99.8	99.54	99.34
KP491	1536	*Klebsiella pneumoniae* strain DSM 30104 16S ribosomal RNA, partial sequence (OM403526.1)	99.8	99.54	99.34
KP590	1536	*Klebsiella pneumoniae* strain DSM 30104 16S ribosomal RNA, partial sequence (OM403526.1)	99.8	99.54	99.34
KP122	1063	*Klebsiella pneumoniae* strain DSM 30104 16S ribosomal RNA, partial sequence (OM403526.1)	99.34	99.53	98.87
KP127	1063	*Klebsiella pneumoniae* strain DSM 30104 16S ribosomal RNA, partial sequence (OM403526.1)	99.34	99.53	98.87
KP148	1063	*Klebsiella pneumoniae* strain DSM 30104 16S ribosomal RNA, partial sequence (OM403526.1)	99.34	99.53	98.87
KP165	1063	*Klebsiella pneumoniae* strain DSM 30104 16S ribosomal RNA, partial sequence (OM403526.1)	99.34	99.53	98.87
KP193	1063	*Klebsiella pneumoniae* strain DSM 30104 16S ribosomal RNA, partial sequence (OM403526.1)	99.34	99.53	98.87

KP236	1063	*Klebsiella pneumoniae* strain DSM 30104 16S ribosomal RNA, partial sequence (OM403526.1)	99.34	99.53	98.87
KP242	1063	*Klebsiella pneumoniae* strain DSM 30104 16S ribosomal RNA, partial sequence (OM403526.1)	99.34	99.53	98.87
KP243	1063	*Klebsiella pneumoniae* strain DSM 30104 16S ribosomal RNA, partial sequence (OM403526.1)	99.34	99.53	98.87
KP247	1063	*Klebsiella pneumoniae* strain DSM 30104 16S ribosomal RNA, partial sequence (OM403526.1)	99.34	99.53	98.87
KP257	1063	*Klebsiella pneumoniae* strain DSM 30104 16S ribosomal RNA, partial sequence (OM403526.1)	99.34	99.53	98.87
KP268	1063	*Klebsiella pneumoniae* strain DSM 30104 16S ribosomal RNA, partial sequence (OM403526.1)	99.34	99.53	98.87
KP305	1063	*Klebsiella pneumoniae* strain DSM 30104 16S ribosomal RNA, partial sequence (OM403526.1)	99.34	99.53	98.87
KP306	1063	*Klebsiella pneumoniae* strain DSM 30104 16S ribosomal RNA, partial sequence (OM403526.1)	99.34	99.53	98.87
KP314	1063	*Klebsiella pneumoniae* strain DSM 30104 16S ribosomal RNA, partial sequence (OM403526.1)	99.34	99.53	98.87
KP324	1063	*Klebsiella pneumoniae* strain DSM 30104 16S ribosomal RNA, partial sequence (OM403526.1)	99.34	99.53	98.87
KP339	1063	*Klebsiella pneumoniae* strain DSM 30104 16S ribosomal RNA, partial sequence (OM403526.1)	99.34	99.53	98.87

KP341	1063	*Klebsiella pneumoniae* strain DSM 30104 16S ribosomal RNA, partial sequence (OM403526.1)	99.34	99.53	98.87
KP355	1063	*Klebsiella pneumoniae* strain DSM 30104 16S ribosomal RNA, partial sequence (OM403526.1)	99.34	99.53	98.87
KP366	1063	*Klebsiella pneumoniae* strain DSM 30104 16S ribosomal RNA, partial sequence (OM403526.1)	99.34	99.53	98.87
KP401	1063	*Klebsiella pneumoniae* strain DSM 30104 16S ribosomal RNA, partial sequence (OM403526.1)	99.34	99.53	98.87
KP428	1063	*Klebsiella pneumoniae* strain DSM 30104 16S ribosomal RNA, partial sequence (OM403526.1)	99.34	99.53	98.87
KP453	1063	*Klebsiella pneumoniae* strain DSM 30104 16S ribosomal RNA, partial sequence (OM403526.1)	99.34	99.53	98.87
KP467	1063	*Klebsiella pneumoniae* strain DSM 30104 16S ribosomal RNA, partial sequence (OM403526.1)	99.34	99.53	98.87
KP480	1063	*Klebsiella pneumoniae* strain DSM 30104 16S ribosomal RNA, partial sequence (OM403526.1)	99.34	99.53	98.87
KP488	1063	*Klebsiella pneumoniae* strain DSM 30104 16S ribosomal RNA, partial sequence (OM403526.1)	99.34	99.53	98.87
KP501	1063	*Klebsiella pneumoniae* strain DSM 30104 16S ribosomal RNA, partial sequence (OM403526.1)	99.34	99.53	98.87
KP510	1063	*Klebsiella pneumoniae* strain DSM 30104 16S ribosomal RNA, partial sequence (OM403526.1)	99.34	99.53	98.87

KP52	1063	*Klebsiella pneumoniae* strain DSM 30104 16S ribosomal RNA, partial sequence (OM403526.1)	99.34	99.53	98.87
KP521	1063	*Klebsiella pneumoniae* strain DSM 30104 16S ribosomal RNA, partial sequence (OM403526.1)	99.34	99.53	98.87
KP525	1063	*Klebsiella pneumoniae* strain DSM 30104 16S ribosomal RNA, partial sequence (OM403526.1)	99.34	99.53	98.87
KP558	1063	*Klebsiella pneumoniae* strain DSM 30104 16S ribosomal RNA, partial sequence (OM403526.1)	99.34	99.53	98.87
KP560	1063	*Klebsiella pneumoniae* strain DSM 30104 16S ribosomal RNA, partial sequence (OM403526.1)	99.34	99.53	98.87
KP599	1063	*Klebsiella pneumoniae* strain DSM 30104 16S ribosomal RNA, partial sequence (OM403526.1)	99.34	99.53	98.87
KP167	1536	*Klebsiella pneumoniae* strain DSM 30104 16S ribosomal RNA, partial sequence (OM403526.1)	99.54	99.48	99.02
KP272	1536	*Klebsiella pneumoniae* strain DSM 30104 16S ribosomal RNA, partial sequence (OM403526.1)	99.54	99.48	99.02
KP455	1536	*Klebsiella pneumoniae* strain DSM 30104 16S ribosomal RNA, partial sequence (OM403526.1)	99.54	99.48	99.02
KP287	1536	*Klebsiella pneumoniae* strain DSM 30104 16S ribosomal RNA, partial sequence (OM403526.1)	99.8	99.41	99.21
KP392	1536	*Klebsiella pneumoniae* strain DSM 30104 16S ribosomal RNA, partial sequence (OM403526.1)	99.8	99.41	99.21

KP125	1277	*Klebsiella pneumoniae* strain DSM 30104 16S ribosomal RNA, partial sequence (OM403526.1)	99.45	99.21	98.66
KP124	1128	*Klebsiella pneumoniae* strain ATCC 13883 16S ribosomal RNA, partial sequence (NR_114506.1)	98.94	99.28	98.23
KP248	1128	*Klebsiella pneumoniae* strain ATCC 13883 16S ribosomal RNA, partial sequence (NR_114506.1)	98.94	99.46	98.41
KP295	1128	*Klebsiella pneumoniae* strain ATCC 13883 16S ribosomal RNA, partial sequence (NR_114506.1)	98.94	99.28	98.23
KP320	1128	*Klebsiella pneumoniae* strain ATCC 13883 16S ribosomal RNA, partial sequence (NR_114506.1)	98.94	99.46	98.41
KP353	1128	*Klebsiella pneumoniae* strain ATCC 13883 16S ribosomal RNA, partial sequence (NR_114506.1)	98.94	99.37	98.32
KP433	1128	*Klebsiella pneumoniae* strain ATCC 13883 16S ribosomal RNA, partial sequence (NR_114506.1)	98.94	99.37	98.32
KP435	1128	*Klebsiella pneumoniae* strain ATCC 13883 16S ribosomal RNA, partial sequence (NR_114506.1)	98.94	99.55	98.49
KP503	1130	*Klebsiella pneumoniae* strain ATCC 13883 16S ribosomal RNA, partial sequence (NR_114506.1)	98.94	99.64	98.58
KP294	655	*Klebsiella pneumoniae* subsp. *rhinoscleromatis* strain R-70 16S ribosomal RNA gene, partial sequence (NR_037084.1)	100	98.97	98.97
KP136	1128	*Klebsiella pneumoniae* subsp. *rhinoscleromatis* ATCC 13884	98.94	99.37	98.32

		16S ribosomal RNA, partial sequence (NR_114507.1)			
KP413	1128	*Klebsiella pneumoniae* subsp. *rhinoscleromatis* ATCC 13884 16S ribosomal RNA, partial sequence (NR_114507.1)	98.94	99.46	98.41
KP70	1208	*Klebsiella pneumoniae* subsp. *rhinoscleromatis* ATCC 13884 16S ribosomal RNA, partial sequence (NR_114507.1)	100	99.75	99.75
KP463	1536	*Klebsiella aerogenes* KCTC 2190 16S ribosomal RNA, complete sequence (NR_102493.2)	100	99.94	99.94

Table S2. Species classification of the *Klebsiella* isolates by average nucleotide identity (ANI) analysis.

Strains	Reference sequences (Accession No.)	ANI (%)
KP553	*K. variicola* subsp *variicola* (CP072130)	99
KP538	*K. variicola* subsp *variicola* (CP072130)	98.99
KP307	*K. variicola* subsp *variicola* (CP072130)	98.98
KP94	*K. variicola* subsp *variicola* (CP072130)	99.01
KP580	*K. variicola* subsp *variicola* (CP072130)	99.04
KP576	*K. variicola* subsp *variicola* (CP072130)	99.01
KP325	*K. variicola* subsp *variicola* (CP072130)	99.07
KP141	*K. variicola* subsp *variicola* (CP072130)	99.07
KP136	*K. variicola* subsp *variicola* (CP072130)	98.78
KP125	*K. variicola* subsp *variicola* (CP072130)	99.01
KP124	*K. variicola* subsp *variicola* (CP072130)	98.77
KP413	*K. quasipneumoniae* subsp *similipneumoniae* (CP084787)	99.18
KP345	*K. quasipneumoniae* subsp *similipneumoniae* (CP084787)	99.15
KP295	*K. quasipneumoniae* subsp *similipneumoniae* (CP084787)	99.22
KP61	*K. pneumoniae* (CP003200)	99.75
KP389	*K. pneumoniae* (CP003200)	99.74
KP20	*K. pneumoniae* (CP003200)	99.75
KP165	*K. pneumoniae* (CP003200)	99.76
KP16	*K. pneumoniae* (CP003200)	99.78
KP127	*K. pneumoniae* (CP003200)	99.75
KP122	*K. pneumoniae* (CP003200)	99.76
KP80	*K. pneumoniae* (CP003200)	99.76
KP67	*K. pneumoniae* (CP003200)	99.79
KP599	*K. pneumoniae* (CP003200)	99.76
KP598	*K. pneumoniae* (CP003200)	99.07
KP593	*K. pneumoniae* (CP003200)	99.68
KP590	*K. pneumoniae* (CP003200)	99.04
KP585	*K. pneumoniae* (CP003200)	99.04
KP579	*K. pneumoniae* (CP003200)	99.75
KP565	*K. pneumoniae* (CP003200)	99.76
KP561	*K. pneumoniae* (CP003200)	99.76
KP560	*K. pneumoniae* (CP003200)	99.74
KP558	*K. pneumoniae* (CP003200)	99.75
KP537	*K. pneumoniae* (CP003200)	98.98
KP525	*K. pneumoniae* (CP003200)	99.75
KP521	*K. pneumoniae* (CP003200)	99.79
KP510	*K. pneumoniae* (CP003200)	99.74
KP501	*K. pneumoniae* (CP003200)	99.78
KP494	*K. pneumoniae* (CP003200)	99.05
KP491	*K. pneumoniae* (CP003200)	99.04

KP488	*K. pneumoniae* (CP003200)	99.78
KP483	*K. pneumoniae* (CP003200)	99.07
KP480	*K. pneumoniae* (CP003200)	99.77
KP467	*K. pneumoniae* (CP003200)	99.73
KP461	*K. pneumoniae* (CP003200)	99.75
KP453	*K. pneumoniae* (CP003200)	99.74
KP450	*K. pneumoniae* (CP003200)	99.06
KP446	*K. pneumoniae* (CP003200)	99.07
KP445	*K. pneumoniae* (CP003200)	99.05
KP443	*K. pneumoniae* (CP003200)	99.09
KP432	*K. pneumoniae* (CP003200)	99.77
KP431	*K. pneumoniae* (CP003200)	99.1
KP430	*K. pneumoniae* (CP003200)	99.76
KP428	*K. pneumoniae* (CP003200)	99.74
KP423	*K. pneumoniae* (CP003200)	99.75
KP408	*K. pneumoniae* (CP003200)	99.76
KP404	*K. pneumoniae* (CP003200)	99.75
KP401	*K. pneumoniae* (CP003200)	99.77
KP388	*K. pneumoniae* (CP003200)	99.75
KP387	*K. pneumoniae* (CP003200)	99.03
KP385	*K. pneumoniae* (CP003200)	99.76
KP377	*K. pneumoniae* (CP003200)	99.75
KP366	*K. pneumoniae* (CP003200)	99.78
KP365	*K. pneumoniae* (CP003200)	99
KP360	*K. pneumoniae* (CP003200)	99.78
KP359	*K. pneumoniae* (CP003200)	99.74
KP357	*K. pneumoniae* (CP003200)	99.19
KP355	*K. pneumoniae* (CP003200)	99.78
KP354	*K. pneumoniae* (CP003200)	99.81
KP341	*K. pneumoniae* (CP003200)	99.76
KP340	*K. pneumoniae* (CP003200)	99.74
KP339	*K. pneumoniae* (CP003200)	99.74
KP332	*K. pneumoniae* (CP003200)	99.75
KP324	*K. pneumoniae* (CP003200)	99.74
KP320	*K. pneumoniae* (CP003200)	99.03
KP315	*K. pneumoniae* (CP003200)	99.76
KP314	*K. pneumoniae* (CP003200)	99.76
KP305	*K. pneumoniae* (CP003200)	99.75
KP294	*K. pneumoniae* (CP003200)	98.95
KP288	*K. pneumoniae* (CP003200)	99.78
KP287	*K. pneumoniae* (CP003200)	99.12
KP283	*K. pneumoniae* (CP003200)	99.77
KP279	*K. pneumoniae* (CP003200)	99.74
KP268	*K. pneumoniae* (CP003200)	99.75

KP266	*K. pneumoniae* (CP003200)	99.75
KP261	*K. pneumoniae* (CP003200)	99.78
KP260	*K. pneumoniae* (CP003200)	99.76
KP257	*K. pneumoniae* (CP003200)	99.76
KP256	*K. pneumoniae* (CP003200)	99.74
KP253	*K. pneumoniae* (CP003200)	99.02
KP247	*K. pneumoniae* (CP003200)	99.78
KP243	*K. pneumoniae* (CP003200)	99.79
KP242	*K. pneumoniae* (CP003200)	99.78
KP236	*K. pneumoniae* (CP003200)	99.75
KP226	*K. pneumoniae* (CP003200)	99.71
KP202	*K. pneumoniae* (CP003200)	99.71
KP186	*K. pneumoniae* (CP003200)	99.74
KP169	*K. pneumoniae* (CP003200)	99.13
KP167	*K. pneumoniae* (CP003200)	99.12
KP166	*K. pneumoniae* (CP003200)	99.7
KP159	*K. pneumoniae* (CP003200)	99.68
KP153	*K. pneumoniae* (CP003200)	99.74
KP149	*K. pneumoniae* (CP003200)	99.74
KP148	*K. pneumoniae* (CP003200)	99.76
KP120	*K. pneumoniae* (CP003200)	99.76
KP117	*K. pneumoniae* (CP003200)	99.7
KP112	*K. pneumoniae* (CP003200)	99.76
KP9	*K. pneumoniae* (CP003200)	99.74
KP70	*K. pneumoniae* (CP003200)	99.14
KP592	*K. pneumoniae* (CP003200)	99.06
KP588	*K. pneumoniae* (CP003200)	99.11
KP586	*K. pneumoniae* (CP003200)	99.13
KP583	*K. pneumoniae* (CP003200)	99.11
KP569	*K. pneumoniae* (CP003200)	99.03
KP567	*K. pneumoniae* (CP003200)	99.16
KP52	*K. pneumoniae* (CP003200)	99.25
KP514	*K. pneumoniae* (CP003200)	98.99
KP503	*K. pneumoniae* (CP003200)	98.97
KP50	*K. pneumoniae* (CP003200)	99.09
KP495	*K. pneumoniae* (CP003200)	98.99
KP472	*K. pneumoniae* (CP003200)	99.1
KP470	*K. pneumoniae* (CP003200)	99.04
KP459	*K. pneumoniae* (CP003200)	99.07
KP455	*K. pneumoniae* (CP003200)	99.1
KP452	*K. pneumoniae* (CP003200)	99.11
KP437	*K. pneumoniae* (CP003200)	99.08
KP435	*K. pneumoniae* (CP003200)	99.06
KP433	*K. pneumoniae* (CP003200)	99.13

KP410	*K. pneumoniae* (CP003200)	99.11
KP399	*K. pneumoniae* (CP003200)	99.07
KP394	*K. pneumoniae* (CP003200)	99.04
KP393	*K. pneumoniae* (CP003200)	98.99
KP392	*K. pneumoniae* (CP003200)	99.16
KP384	*K. pneumoniae* (CP003200)	98.97
KP382	*K. pneumoniae* (CP003200)	99.13
KP363	*K. pneumoniae* (CP003200)	99.01
KP36	*K. pneumoniae* (CP003200)	99.88
KP356	*K. pneumoniae* (CP003200)	99.14
KP353	*K. pneumoniae* (CP003200)	99.08
KP352	*K. pneumoniae* (CP003200)	98.99
KP329	*K. pneumoniae* (CP003200)	99.04
KP306	*K. pneumoniae* (CP003200)	99.74
KP302	*K. pneumoniae* (CP003200)	99.79
KP301	*K. pneumoniae* (CP003200)	99.19
KP298	*K. pneumoniae* (CP003200)	99.02
KP297	*K. pneumoniae* (CP003200)	99.07
KP284	*K. pneumoniae* (CP003200)	99
KP281	*K. pneumoniae* (CP003200)	99.12
KP276	*K. pneumoniae* (CP003200)	99.11
KP272	*K. pneumoniae* (CP003200)	99.05
KP270	*K. pneumoniae* (CP003200)	99.1
KP259	*K. pneumoniae* (CP003200)	99.06
KP255	*K. pneumoniae* (CP003200)	99.72
KP248	*K. pneumoniae* (CP003200)	99.07
KP246	*K. pneumoniae* (CP003200)	99.05
KP241	*K. pneumoniae* (CP003200)	99.13
KP233	*K. pneumoniae* (CP003200)	99.02
KP231	*K. pneumoniae* (CP003200)	98.99
KP227	*K. pneumoniae* (CP003200)	99.11
KP220	*K. pneumoniae* (CP003200)	99.04
KP214	*K. pneumoniae* (CP003200)	99.02
KP208	*K. pneumoniae* (CP003200)	99.75
KP193	*K. pneumoniae* (CP003200)	99.8
KP181	*K. pneumoniae* (CP003200)	99.8
KP115	*K. pneumoniae* (CP003200)	99.12
KP113	*K. pneumoniae* (CP003200)	99.02
KP463	*K. aerogenes* (FKIV00000000)	98.66

Table S3. The sequence types (STs) of *K. pneumoniae*.

Strains	ST	*gapA*	*infB*	*mdh*	*pgi*	*phoE*	*rpoB*	*tonB*
KP537	1	4	4	1	1	7	4	10
KP297	3	5	5	1	1	9	6	11
KP61	11	3	3	1	1	1	1	4
KP389	11	3	3	1	1	1	1	4
KP20	11	3	3	1	1	1	1	4
KP165	11	3	3	1	1	1	1	4
KP16	11	3	3	1	1	1	1	4
KP9	11	3	3	1	1	1	1	4
KP80	11	3	3	1	1	1	1	4
KP67	11	3	3	1	1	1	1	4
KP599	11	3	3	1	1	1	1	4
KP593	11	3	3	1	1	1	1	4
KP579	11	3	3	1	1	1	1	4
KP565	11	3	3	1	1	1	1	4
KP561	11	3	3	1	1	1	1	4
KP560	11	3	3	1	1	1	1	4
KP558	11	3	3	1	1	1	1	4
KP525	11	3	3	1	1	1	1	4
KP521	11	3	3	1	1	1	1	4
KP510	11	3	3	1	1	1	1	4
KP501	11	3	3	1	1	1	1	4
KP488	11	3	3	1	1	1	1	4
KP480	11	3	3	1	1	1	1	4
KP467	11	3	3	1	1	1	1	4
KP461	11	3	3	1	1	1	1	4
KP453	11	3	3	1	1	1	1	4
KP432	11	3	3	1	1	1	1	4
KP430	11	3	3	1	1	1	1	4
KP428	11	3	3	1	1	1	1	4
KP423	11	3	3	1	1	1	1	4
KP408	11	3	3	1	1	1	1	4
KP404	11	3	3	1	1	1	1	4
KP401	11	3	3	1	1	1	1	4
KP388	11	3	3	1	1	1	1	4
KP385	11	3	3	1	1	1	1	4
KP377	11	3	3	1	1	1	1	4
KP366	11	3	3	1	1	1	1	4
KP360	11	3	3	1	1	1	1	4
KP36	11	3	3	1	1	1	1	4
KP359	11	3	3	1	1	1	1	4
KP355	11	3	3	1	1	1	1	4
KP354	11	3	3	1	1	1	1	4

KP341	11	3	3	1	1	1	1	4
KP340	11	3	3	1	1	1	1	4
KP339	11	3	3	1	1	1	1	4
KP332	11	3	3	1	1	1	1	4
KP324	11	3	3	1	1	1	1	4
KP315	11	3	3	1	1	1	1	4
KP314	11	3	3	1	1	1	1	4
KP306	11	3	3	1	1	1	1	4
KP305	11	3	3	1	1	1	1	4
KP302	11	3	3	1	1	1	1	4
KP288	11	3	3	1	1	1	1	4
KP283	11	3	3	1	1	1	1	4
KP279	11	3	3	1	1	1	1	4
KP268	11	3	3	1	1	1	1	4
KP261	11	3	3	1	1	1	1	4
KP260	11	3	3	1	1	1	1	4
KP257	11	3	3	1	1	1	1	4
KP256	11	3	3	1	1	1	1	4
KP255	11	3	3	1	1	1	1	4
KP247	11	3	3	1	1	1	1	4
KP243	11	3	3	1	1	1	1	4
KP242	11	3	3	1	1	1	1	4
KP236	11	3	3	1	1	1	1	4
KP226	11	3	3	1	1	1	1	4
KP208	11	3	3	1	1	1	1	4
KP202	11	3	3	1	1	1	1	4
KP193	11	3	3	1	1	1	1	4
KP186	11	3	3	1	1	1	1	4
KP181	11	3	3	1	1	1	1	4
KP166	11	3	3	1	1	1	1	4
KP159	11	3	3	1	1	1	1	4
KP153	11	3	3	1	1	1	1	4
KP149	11	3	3	1	1	1	1	4
KP148	11	3	3	1	1	1	1	4
KP120	11	3	3	1	1	1	1	4
KP117	11	3	3	1	1	1	1	4
KP112	11	3	3	1	1	1	1	4
KP266	11	3	3	1	1	1	1	4
KP393	15	1	1	1	1	1	1	1
KP329	15	1	1	1	1	1	1	1
KP598	15	1	1	1	1	1	1	1
KP472	17	2	1	1	1	4	4	4
KP284	17	2	1	1	1	4	4	4
KP494	23	2	1	1	1	9	4	12

KP491	23	2	1	1	1	9	4	12
KP483	23	2	1	1	1	9	4	12
KP446	23	2	1	1	1	9	4	12
KP445	23	2	1	1	1	9	4	12
KP387	23	2	1	1	1	9	4	12
KP590	23	2	1	1	1	9	4	12
KP459	23	2	1	1	1	9	4	12
KP437	23	2	1	1	1	9	4	12
KP384	23	2	1	1	1	9	4	12
KP352	23	2	1	1	1	9	4	12
KP231	23	2	1	1	1	9	4	12
KP113	23	2	1	1	1	9	4	12
KP435	29	2	3	2	2	6	4	4
KP503	35	2	1	2	1	10	1	19
KP588	36	2	1	2	1	7	1	7
KP583	37	2	9	2	1	13	1	16
KP52	37	2	9	2	1	13	1	16
KP357	37	2	9	2	1	13	1	16
KP220	37	2	9	2	1	13	1	16
KP455	45	2	1	1	6	7	1	12
KP431	45	2	1	1	6	7	1	12
KP167	45	2	1	1	6	7	1	12
KP70	55	2	1	1	17	7	16	4
KP470	65	2	1	2	1	10	4	13
KP394	65	2	1	2	1	10	4	13
KP567	86	9	4	2	1	1	1	27
KP433	86	9	4	2	1	1	1	27
KP392	104	2	3	1	1	2	1	43
KP320	107	2	1	2	17	27	1	39
KP214	111	2	1	5	1	17	4	42
KP365	133	12	1	1	2	5	1	36
KP301	221	2	5	1	6	9	1	10
KP233	299	2	10	1	1	56	24	31
KP127	340	3	3	1	1	1	1	18
KP122	340	3	3	1	1	1	1	18
KP443	392	3	4	6	1	7	4	40
KP50	412	2	1	2	1	9	1	112
KP452	412	2	1	2	1	9	1	112
KP410	412	2	1	2	1	9	1	112
KP399	412	2	1	2	1	9	1	112
KP356	412	2	1	2	1	9	1	112
KP276	412	2	1	2	1	9	1	112
KP246	412	2	1	2	1	9	1	112
KP241	412	2	1	2	1	9	1	112

KP115	412	2	1	2	1	9	1	112
KP294	423	2	9	2	65	13	1	16
KP287	423	2	9	2	65	13	1	16
KP450	656	4	4	1	1	7	4	4
KP298	656	4	4	1	1	7	4	4
KP169	685	2	1	2	1	3	4	25
KP270	687	2	3	10	1	20	4	110
KP272	700	10	1	17	37	12	1	9
KP248	828	2	1	1	26	1	1	54
KP514	1049	2	3	4	97	12	1	39
KP363	1049	2	3	4	97	12	1	39
KP281	1128	2	1	1	1	7	1	4
KP227	1569	10	1	11	1	9	8	257
KP586	2159	4	7	1	37	177	4	6
KP592	2165	2	1	1	1	1	4	336
KP382	2264	3	9	47	1	13	1	340
KP353	3509	9	4	2	134	1	1	27
KP259	6013	4	1	1	26	3	7	24
KP495	6023	2	3	2	1	1	4	840
KP585	6026	2	1	1	82	1	4	336
KP253	6254	2	6	1	401	4	1	6
KP569	6255	2	1	1	46	600	1	12

Table S4. Minimum inhibitory concentrations (MICs) of 11 antimicrobials (μg/mL) against 96 integron-positive isolates.

Strains	Amikacin (≥64)	Gentamicin (≥16)	Aztreonam (≥16)	Cefepime (≥16)	Ceftazidime (≥16)	Chloramphenicol (≥32)	Fosfomycin (≥256)	Meropenem (≥4)	Nalidixic Acid (≥32)	Tetracycline (≥16)	Tigecycline (≥8)
KP112	128	128	128	128	128	16	512	128	128	512	32
KP117	128	128	128	128	128	512	512	128	512	512	8
KP120	128	128	128	128	128	8	512	128	512	512	4
KP122	1	0.25	8	4	0.5	256	512	0.25	16	2	2
KP127	4	64	128	64	64	4	512	128	512	256	4
KP148	0.5	0.25	0.5	0.25	8	8	512	0.25	32	16	8
KP149	0.5	0.25	128	64	128	8	256	8	16	16	4
KP153	128	128	128	128	128	32	512	128	512	8	1
KP159	2	0.25	128	128	64	8	256	128	128	32	8
KP16	2	1	0.25	0.25	0.25	32	512	0.25	4	32	4
KP165	4	1	8	16	2	64	512	0.25	256	256	2
KP166	128	128	64	32	8	128	128	0.25	256	64	4
KP167	8	16	128	128	64	8	256	128	512	8	4
KP169	64	128	32	128	128	16	128	16	128	32	16
KP186	1	32	32	128	128	256	64	8	512	512	0.5
KP20	128	128	128	128	128	8	256	128	512	8	1
KP202	128	128	128	128	128	1	64	128	512	128	0.5
KP226	128	128	128	128	128	32	512	128	512	512	8
KP236	1	0.25	1	128	0.5	512	64	64	32	512	32
KP242	128	128	128	128	64	512	256	128	512	8	2
KP243	128	128	128	128	128	8	512	128	512	8	2
KP247	128	128	128	128	64	64	256	128	512	16	4
KP253	128	128	128	128	128	16	512	128	512	512	8

KP256	1	0.25	0.25	0.25	0.25	4	64	0.25	8	1	1
KP257	0.5	0.25	64	16	16	2	64	0.25	8	1	0.5
KP260	128	128	128	128	128	512	128	128	512	256	4
KP261	1	0.25	0.25	0.25	0.25	2	64	0.25	4	1	0.5
KP266	128	128	128	128	128	2	512	128	512	256	2
KP268	128	128	128	128	128	1	64	128	512	256	0.5
KP279	128	128	128	128	128	32	512	128	512	512	8
KP283	128	128	128	128	128	4	512	128	512	8	0.5
KP287	1	0.25	128	128	64	32	256	128	512	8	2
KP288	2	0.25	128	8	64	16	32	16	64	128	2
KP294	128	128	128	128	128	512	512	128	512	512	4
KP305	1	0.25	0.25	0.25	0.25	4	32	0.25	4	2	2
KP307	2	0.25	0.25	0.25	0.25	4	64	0.25	8	2	2
KP314	128	128	128	128	128	512	512	128	512	512	16
KP315	4	0.25	128	64	32	16	512	4	512	8	4
KP320	1	128	128	64	128	512	64	0.25	512	256	16
KP324	1	0.25	0.25	0.25	0.5	2	32	0.25	8	2	2
KP332	8	0.25	0.25	0.25	0.25	4	64	0.25	8	2	2
KP339	0.5	0.25	0.25	0.25	0.25	1	32	0.25	8	2	2
KP340	1	0.5	0.25	0.25	0.25	4	8	0.25	4	2	2
KP341	0.5	0.25	128	32	0.25	4	32	0.25	4	256	2
KP354	128	128	128	128	128	512	256	128	512	512	4
KP355	1	0.25	0.25	0.25	0.25	4	256	0.25	4	2	2
KP357	1	0.5	128	128	64	32	256	128	512	8	2
KP359	128	128	128	128	128	512	512	128	512	512	8
KP360	2	32	16	32	32	512	512	0.25	128	512	16

KP365	1	0.25	128	128	32	8	256	128	128	32	8
KP366	128	128	128	128	128	512	512	128	128	512	16
KP377	128	128	128	128	128	512	256	128	512	512	4
KP385	0.5	0.25	0.25	0.25	0.25	4	64	0.25	8	1	1
KP387	128	128	128	128	128	16	512	128	512	8	1
KP388	128	128	128	128	128	32	512	128	512	16	1
KP389	128	128	128	128	64	8	256	128	128	16	8
KP401	128	128	128	128	128	8	256	128	128	16	4
KP404	1	0.25	0.25	0.25	0.25	2	64	0.25	4	1	0.5
KP408	1	0.25	0.25	0.25	0.25	8	16	0.25	64	16	8
KP423	0.5	0.25	0.25	0.25	0.25	4	8	0.25	128	8	4
KP428	1	8	1	1	8	8	64	0.25	512	512	4
KP430	1	0.25	0.25	0.25	0.25	2	8	0.25	4	64	0.5
KP431	128	128	128	128	64	4	512	128	512	8	0.5
KP432	128	128	128	128	64	512	256	128	128	32	8
KP443	8	16	64	128	128	16	512	128	512	512	8
KP445	1	0.25	128	128	128	8	256	128	512	8	2
KP446	32	512	4	64	32	32	128	0.25	0.25	512	512
KP450	8	8	64	128	128	16	256	128	512	512	8
KP453	128	128	128	128	128	512	512	128	512	512	1
KP461	1	0.25	0.25	0.25	0.25	4	64	0.25	4	1	0.5
KP467	1	0.25	0.25	0.25	0.25	4	64	0.25	8	1	0.5
KP480	1	0.25	128	128	128	256	512	128	512	512	1
KP483	1	0.25	128	32	16	8	512	4	512	4	0.5
KP488	128	128	128	32	128	512	512	128	512	512	16
KP491	1	0.25	128	128	64	16	256	128	512	8	2

KP494	128	128	128	128	128	8	512	128	512	512	8
KP501	128	128	128	128	128	512	512	128	512	512	16
KP510	2	0.25	0.25	0.25	1	8	32	0.25	16	8	4
KP521	128	128	128	128	64	512	256	128	512	8	4
KP525	128	128	128	128	128	512	512	128	512	512	16
KP537	1	0.25	128	128	64	8	256	128	512	8	1
KP538	128	128	128	128	128	4	512	128	512	8	0.5
KP553	128	128	128	128	128	512	512	64	512	512	4
KP558	2	0.25	0.25	0.25	0.25	2	32	0.25	8	2	2
KP560	128	128	128	128	128	512	256	128	512	8	4
KP561	4	32	128	64	128	512	256	8	32	8	8
KP565	128	128	128	128	32	16	256	128	128	16	4
KP579	32	0.25	128	128	64	8	256	128	128	16	2
KP585	1	0.25	128	128	64	16	256	128	128	16	16
KP590	1	0.25	128	128	128	16	512	128	512	8	2
KP593	128	128	128	128	128	512	512	128	512	512	8
KP598	128	128	128	128	128	512	512	128	512	512	4
KP599	128	128	128	128	128	512	256	128	128	64	16
KP61	1	0.25	128	128	64	16	512	128	512	128	4
KP67	1	0.25	64	32	32	8	512	16	64	8	4
KP80	128	128	128	128	128	8	256	128	512	8	1
Total	44	52	71	73	70	38	66	67	74	56	29

* The brown represents resistance to the antimicrobials and the yellow represents MDR isolates.

Table S5. Minimum inhibitory concentrations (MICs) of 11 antimicrobials (μg/mL) against 71 integron-negative isolates.

Strains	Amikacin (≥64)	Gentamicin (≥16)	Aztreonam (≥16)	Cefepime (≥16)	Ceftazidime (≥16)	Chloramphenicol (≥32)	Fosfomycin (≥256)	Meropenem (≥4)	Nalidixic Acid (≥32)	Tetracycline (≥16)	Tigecycline (≥8)
KP297	2	32	16	32	4	64	128	0.25	16	128	16
KP281	128	128	128	64	8	8	128	0.25	128	16	16
KP394	32	32	64	32	32	64	32	16	64	64	16
KP241	128	128	128	128	128	512	128	128	512	512	8
KP70	128	128	128	128	128	32	512	128	128	512	32
KP113	128	128	128	128	128	512	512	128	128	4	16
KP329	1	32	32	128	128	8	16	8	32	128	1
KP231	1	64	64	128	128	256	64	64	512	512	0.5
KP233	128	128	128	128	64	512	128	128	512	16	2
KP259	128	128	128	128	128	256	128	128	128	16	2
KP392	128	32	0.25	1	0.5	4	2	0.03	4	2	0.5
KP255	128	128	128	128	128	16	128	64	512	2	1
KP298	128	128	128	128	64	8	64	1	512	8	1
KP246	128	128	128	128	128	512	128	128	512	8	1
KP248	128	128	128	128	64	512	128	128	512	8	1
KP181	128	32	64	32	32	8	256	16	64	8	1
KP284	2	64	2	4	16	512	128	0.25	128	512	4
KP302	128	128	128	128	128	512	128	32	512	256	4
KP393	128	128	128	128	128	512	128	128	512	256	4
KP270	128	128	128	128	128	512	128	128	512	8	4
KP352	64	128	128	128	128	64	128	8	128	8	4
KP214	1	0.25	0.25	0.25	0.25	4	128	0.25	16	128	8

KP141	2	0.25	0.25	0.25	0.25	8	32	0.25	128	16	8
KP514	1	0.25	0.25	0.5	0.5	64	64	0.03	32	64	16
KP503	1	0.25	64	32	32	64	64	16	64	64	32
KP220	1	0.25	0.25	0.25	0.25	4	256	0.25	16	8	16
KP227	1	0.25	0.25	0.25	0.25	4	256	0.25	64	8	8
KP295	0.5	0.25	32	8	8	4	16	0.5	8	64	1
KP272	1	0.5	128	128	128	512	128	128	512	16	2
KP495	1	0.25	0.25	0.25	0.25	8	4	0.25	8	2	1
KP410	1	0.25	0.25	0.25	0.25	4	8	0.25	4	1	0.5
KP470	1	0.5	0.25	0.25	0.25	8	16	0.25	4	2	1
KP384	1	0.25	0.25	0.25	0.25	8	32	0.25	4	2	1
KP435	1	0.25	0.25	0.25	0.25	8	32	0.25	4	2	1
KP459	1	0.25	0.25	0.25	0.25	8	32	0.25	8	2	0.5
KP586	1	0.5	0.25	0.25	0.25	8	32	0.25	4	2	1
KP382	0.5	0.25	0.25	0.25	0.25	8	32	0.25	8	2	1
KP463	1	0.25	0.25	0.25	0.25	8	32	0.25	4	2	1
KP276	0.5	0.25	0.25	0.25	0.25	4	64	0.25	4	1	1
KP433	1	0.25	0.25	0.25	0.25	8	64	0.25	4	2	1
KP437	1	0.25	0.25	0.25	0.25	8	64	0.25	4	2	1
KP452	1	0.25	0.25	0.25	0.25	8	64	0.25	4	2	1
KP472	1	0.25	0.25	0.25	0.25	4	64	0.25	4	2	1
KP567	2	0.5	0.125	0.125	0.125	4	64	0.03	8	2	2
KP569	1	0.25	0.25	0.25	0.25	8	64	0.25	8	2	1
KP583	1	0.25	0.25	0.25	0.25	8	64	0.25	8	2	1
KP588	1	0.25	0.25	0.25	0.25	4	64	0.25	4	2	1
KP306	0.5	0.25	0.25	0.25	0.25	8	64	0.25	8	2	1

KP325	1	0.5	0.25	0.25	0.25	8	64	0.25	8	2	1
KP345	0.5	0.25	0.25	0.25	0.25	8	64	0.25	8	2	1
KP413	0.5	0.25	0.25	0.25	0.25	8	64	0.25	8	2	1
KP576	2	0.5	0.25	0.25	0.25	8	64	0.25	4	2	1
KP580	1	0.5	0.25	0.25	0.25	8	64	0.25	8	2	1
KP455	1	0.25	0.25	0.25	0.25	16	64	0.25	4	2	1
KP363	0.5	0.25	64	128	128	8	64	4	8	4	1
KP136	1	0.25	0.25	0.25	0.25	8	32	0.25	128	4	2
KP193	1	0.25	0.25	0.25	0.25	4	256	0.25	8	8	2
KP399	1	0.5	0.25	0.25	0.25	4	32	0.25	16	128	4
KP353	1	0.25	128	32	32	8	128	16	16	16	4
KP301	1	0.25	0.5	128	64	8	32	0.25	32	512	4
KP115	1	0.25	128	128	32	16	256	128	128	16	4
KP124	2	0.25	0.25	0.25	0.25	8	64	0.25	64	4	4
KP592	2	0.25	0.25	0.25	0.25	8	8	0.25	16	8	4
KP94	2	0.25	0.25	0.25	0.25	8	8	0.25	16	8	4
KP125	1	0.25	0.25	0.25	0.25	8	32	0.25	8	8	4
KP208	1	0.25	0.25	0.25	0.25	8	512	0.25	8	8	4
KP356	0.5	0.25	128	64	128	8	128	8	32	8	4
KP36	1	0.25	128	128	64	8	256	128	512	8	4
KP50	1	0.25	128	128	64	8	256	128	512	8	4
KP9	1	0.25	128	128	128	8	512	128	512	8	4
KP52	1	0.25	128	128	64	8	512	128	512	8	4
Total	16	21	30	30	29	18	12	26	33	22	12

* The brown represents resistance to the antimicrobials and the yellow represents MDR isolates.

Table S6. The 143 types of drug resistance genes annotated in the genomes of the 167 *Klebsiella* isolates.

Resistance genes	Drug Classes	Resistance Mechanism	Integron-positive isolates (n=96)	Integron-negative isolates (n=71)
mdtA	aminocoumarin	antibiotic efflux	1	1
mdtB	aminocoumarin	antibiotic efflux	95	71
mdtC	aminocoumarin	antibiotic efflux	96	71
crcB	aminocoumarin	antibiotic efflux	1	10
baeR	aminocoumarin, aminoglycoside	antibiotic efflux	96	71
cpxA	aminocoumarin, aminoglycoside	antibiotic efflux	96	71
aac(3)-IId	aminoglycoside	antibiotic inactivation	6	2
aadA2	aminoglycoside	antibiotic inactivation	72	4
aadA5	aminoglycoside	antibiotic inactivation	15	3
acrD	aminoglycoside	antibiotic efflux	96	71
aph(3')-Ia	aminoglycoside	antibiotic inactivation	4	1
aph(3'')-Ib	aminoglycoside	antibiotic inactivation	5	6
aph(6)-Id	aminoglycoside	antibiotic inactivation	8	7
kdpE	aminoglycoside	antibiotic efflux	96	71
rmtB	aminoglycoside	antibiotic target alteration	53	5
Kpne_kpnE	aminoglycoside, rifamycin, macrolide, peptide, cephalosporin, tetracycline	antibiotic efflux	96	71
Kpne_KpnF	aminoglycoside, rifamycin, macrolide, peptide, cephalosporin, tetracycline	antibiotic efflux	96	71
Escherichia coli mdfA	benzalkonium chloride, tetracycline, rhodamine	antibiotic efflux	94	71
lptD	carbapenem, peptides, aminocoumarin, rifamycin	antibiotic efflux	96	71

bla$_{SHV-142}$	carbapenem, penam, cephalosporin	antibiotic inactivation	64	30
bla$_{SHV-33}$	carbapenem, penam, cephalosporin	antibiotic inactivation	1	3
bla$_{SHV-66}$	carbapenem, penam, cephalosporin	antibiotic inactivation	17	2
bla$_{SHV-94}$	carbapenem, penam, cephalosporin	antibiotic inactivation	1	1
bla$_{CTX-M-14}$	cephalosporin	antibiotic inactivation	17	5
bla$_{CTX-M-15}$	cephalosporin	antibiotic inactivation	4	2
bla$_{CTX-M-27}$	cephalosporin	antibiotic inactivation	2	1
bla$_{CTX-M-3}$	cephalosporin	antibiotic inactivation	3	1
bla$_{CTX-M-65}$	cephalosporin	antibiotic inactivation	48	4
bla$_{DHA-1}$	cephalosporin, cephamycin	antibiotic inactivation	4	1
dfrA17	diaminopyrimidine	antibiotic target replacement	1	1
rsmA	diaminopyrimidine, fluoroquinolone, phenicol	antibiotic efflux	96	71
oqxA	diaminopyrimidine, glycylcycline, tetracycline , nitrofuran, fluoroquinolone	antibiotic efflux	41	64
oqxB	diaminopyrimidine, glycylcycline, tetracycline , nitrofuran, fluoroquinolone	antibiotic efflux	41	63
qacEΔ1	disinfecting agents and antiseptics	antibiotic efflux	77	5
Ecol_gyrA_TRC	fluoroquinolone	antibiotic target alteration	6	4
Ecol_parC_FLO	fluoroquinolone	antibiotic target alteration	78	12
emrB	fluoroquinolone	antibiotic efflux	86	48
emrR	fluoroquinolone	antibiotic efflux	93	71
mdtH	fluoroquinolone	antibiotic efflux	96	71
mdtK	fluoroquinolone	antibiotic efflux	95	70
qnrB4	fluoroquinolone	antibiotic target protection	3	1

qnrS1	fluoroquinolone	antibiotic target protection	54	11
aac(6')-Ib-cr	fluoroquinolone, aminoglycoside	antibiotic inactivation	10	2
Ecol_uhpT_MULT	fosfomycin	antibiotic target alteration	96	71
fosA6	fosfomycin	antibiotic inactivation	95	57
mphA	macrolide	antibiotic inactivation	23	7
msbA	nitroimidazole	antibiotic efflux	96	71
$bla_{\text{SHV-103}}$	penam, carbapenem, cephalosporin	antibiotic inactivation	6	12
$bla_{\text{KPC-2}}$	penam, carbapenem, cephalosporin, monobactam	antibiotic inactivation	74	12
Kpne_OmpK37	penam, carbapenem, penem, cephalosporin, cephamycin, monobactam	reduced permeability to antibiotic	95	70
$bla_{\text{OXA-1}}$	penam, cephalosporin	antibiotic inactivation	3	2
Ecol_ampH_BLA	penam, cephalosporin	antibiotic inactivation	96	71
acrF	penam, cephalosporin, cephamycin, fluoroquinolone	antibiotic efflux	87	50
hns	penam, macrolide, cephalosporin, cephamycin, tetracycline, fluoroquinolone	antibiotic efflux	96	71
crp	penam, macrolide, fluoroquinolone	antibiotic efflux	95	71
len-16	penam, penem	antibiotic inactivation	2	2
ompA	penam, penem, carbapenem, cephalosporin, cephamycin, monobactam	reduced permeability to antibiotic	96	71
$bla_{\text{TEM-1}}$	penam, penem, cephalosporin, monobactam	antibiotic inactivation	54	10

Ecol_marR_MULT	penam, triclosan, phenicol, cephalosporin, tetracycline, glycylcycline, rifamycin, fluoroquinolone	antibiotic efflux; antibiotic target alteration	96	70
acrB	penam, triclosan, tetracycline, cephalosporin, glycylcycline, fluoroquinolone, rifamycin , phenicol	antibiotic efflux	96	71
Kpne_acrA	penam, triclosan, tetracycline, cephalosporin, glycylcycline, fluoroquinolone, rifamycin , phenicol	antibiotic efflux	96	71
Kpne_KpnG	penem, cephalosporin, macrolide, carbapenem, aminoglycoside, peptide, penam, fluoroquinolone	antibiotic efflux	95	71
Kpne_KpnH	penem, cephalosporin, macrolide, carbapenem, aminoglycoside, peptide, penam, fluoroquinolone	antibiotic efflux	10	23
tolC	penem, tetracycline, aminocoumarin, cephalosporin, macrolide, carbapenem, aminoglycoside, glycylcycline, rifamycin, peptide, penam, triclosan, cephamycin, fluoroquinolone, phenicol	antibiotic efflux	96	71
Ecol_soxS_MULT	penem, tetracycline, cephalosporin, carbapenem, penam, triclosan, cephamycin, glycylcycline, monobactam, fluoroquinolone, phenicol, rifamycin	antibiotic target alteration; antibiotic efflux; reduced permeability to antibiotic	96	71
marA	penem, tetracycline, cephalosporin, carbapenem, penam, triclosan, cephamycin,	antibiotic efflux; reduced permeability to antibiotic	96	70

	glycylcycline, monobactam, fluoroquinolone, phenicol, rifamycin			
ramA	penem, tetracycline, cephalosporin, carbapenem, penam, triclosan, cephamycin, glycylcycline, monobactam, fluoroquinolone, phenicol, rifamycin	antibiotic efflux; reduced permeability to antibiotic	93	71
arnT	peptide	antibiotic target alteration	96	71
bacA	peptide	antibiotic target alteration	96	71
eptB	peptide	antibiotic target alteration	96	71
pmrF	peptide	antibiotic target alteration	96	71
ugd	peptide	antibiotic target alteration	95	72
yojI	peptide	antibiotic efflux	96	71
Ecol_catII	phenicol	antibiotic inactivation	37	2
floR	phenicol	antibiotic efflux	7	4
sul1	sulfonamide	antibiotic target replacement	16	2
sul2	sulfonamide	antibiotic target replacement	44	8
tet(A)	tetracycline	antibiotic efflux	40	12
tet(D)	tetracycline	antibiotic efflux	6	1
Ecol_soxR_MULT	tetracycline, cephalosporin, penam, triclosan, glycylcycline, fluoroquinolone, phenicol, rifamycin	antibiotic target alteration; antibiotic efflux	96	71
lap-2	tetracycline, fluoroquinolone, aminoglycoside, rifamycin	antibiotic inactivation	36	7
bla$_{\text{SHV-1}}$	triclosan	antibiotic target alteration	71	9
aac(3)-IV	aminoglycoside	antibiotic inactivation	1	0
aac(6')-Ib	aminoglycoside	antibiotic inactivation	1	0

aac(6')-Ib10	aminoglycoside	antibiotic inactivation	1	0
aac(6')-Ib9	aminoglycoside	antibiotic inactivation	3	0
aadA16	aminoglycoside	antibiotic inactivation	6	0
ant(2")-Ia	aminoglycoside	antibiotic inactivation	1	0
ant(3")-IIa	aminoglycoside	antibiotic inactivation	1	0
armA	aminoglycoside	antibiotic target alteration	2	0
rmtF	aminoglycoside	antibiotic target alteration	1	0
$bla_{\text{SHV-11}}$	carbapenem, penam, cephalosporin	antibiotic inactivation	1	0
$bla_{\text{SHV-27}}$	carbapenem, penam, cephalosporin	antibiotic inactivation	1	0
$bla_{\text{CTX-M-123}}$	cephalosporin	antibiotic inactivation	1	0
dfrA12	diaminopyrimidine	antibiotic target replacement	3	0
dfrA14	diaminopyrimidine	antibiotic target replacement	3	0
dfrA27	diaminopyrimidine	antibiotic target replacement	2	0
qnrA1	fluoroquinolone	antibiotic target protection	1	0
qnrB1	fluoroquinolone	antibiotic target protection	2	0
qnrB2	fluoroquinolone	antibiotic target protection	3	0
qnrB20	fluoroquinolone	antibiotic target protection	1	0
blmA	glycopeptide	antibiotic inactivation	7	0
mphE	macrolide	antibiotic inactivation	2	0
fona-6	penam	antibiotic inactivation	6	0
$bla_{\text{NDM-1}}$	penam, carbapenem, cephalosporin, cephamycin	antibiotic inactivation	5	0
$bla_{\text{KPC-3}}$	penam, carbapenem, cephalosporin, monobactam	antibiotic inactivation	1	0
$bla_{\text{OXA-10}}$	penam, cephalosporin	antibiotic inactivation	1	0
$bla_{\text{OXA-232}}$	penam, cephalosporin	antibiotic inactivation	1	0

bla$_{\text{NDM-5}}$	penam, cephalosporin, carbapenem, cephamycin	antibiotic inactivation	2	0
len-19	penam, penem	antibiotic inactivation	1	0
bla$_{\text{IMP-4}}$	penam, penem, carbapenem, cephalosporin, cephamycin	antibiotic inactivation	8	0
bla$_{\text{TEM-135}}$	penam, penem, cephalosporin, monobactam	antibiotic inactivation	1	0
mcr-8.1	peptide	antibiotic target alteration	2	0
catB3	phenicol	antibiotic inactivation	2	0
cmlA5	phenicol	antibiotic efflux	1	0
msrE	pleuromutilin, macrolide, oxazolidinone, tetracycline, streptogramin, phenicol, lincosamide	antibiotic target protection	2	0
arr-2	rifamycin	antibiotic inactivation	2	0
arr-3	rifamycin	antibiotic inactivation	10	0
mexG	acridine dye, tetracycline, fluoroquinolone	antibiotic efflux	0	1
aadA23	aminoglycoside	antibiotic inactivation	0	1
bla$_{\text{SHV-187}}$	carbapenem, penam, cephalosporin	antibiotic inactivation	0	2
bla$_{\text{SHV-2}}$	carbapenem, penam, cephalosporin	antibiotic inactivation	0	6
bla$_{\text{SHV-28}}$	carbapenem, penam, cephalosporin	antibiotic inactivation	0	1
bla$_{\text{SHV-66}}$	carbapenem, penam, cephalosporin	antibiotic inactivation	0	1
bla$_{\text{SHV-71}}$	carbapenem, penam, cephalosporin	antibiotic inactivation	0	1
bla$_{\text{CTX-M-55}}$	cephalosporin	antibiotic inactivation	0	2
qacH	fluoroquinolone	antibiotic efflux	0	2
qnrS2	fluoroquinolone	antibiotic target protection	0	1
fosA3	fosfomycin	antibiotic inactivation	0	3
fosA5	fosfomycin	antibiotic inactivation	0	11

bla$_{\text{OKP-B-1}}$	penam, cephalosporin	antibiotic inactivation	0	1
bla$_{\text{OKP-B-2}}$	penam, cephalosporin	antibiotic inactivation	0	1
bla$_{\text{OKP-B-6}}$	penam, cephalosporin	antibiotic inactivation	0	1
len-10	penam, penem	antibiotic inactivation	0	2
len-13	penam, penem	antibiotic inactivation	0	2
len-2	penam, penem	antibiotic inactivation	0	1
len-9	penam, penem	antibiotic inactivation	0	1
bla$_{\text{TEM-116}}$	penam, penem, cephalosporin, monobactam	antibiotic inactivation	0	1
Kpne_acrR_MULT	penam, triclosan, phenicol, cephalosporin, tetracycline, glycylcycline, rifamycin, fluoroquinolone	antibiotic efflux; antibiotic target alteration	0	4
Kpne_ramR	penam, triclosan, phenicol, cephalosporin, tetracycline, glycylcycline, rifamycin, fluoroquinolone	antibiotic efflux; antibiotic target alteration	0	3
erm(42)	streptogramin, macrolide, lincosamide	antibiotic target alteration	0	1
sul3	sulfonamide	antibiotic target replacement	0	2
tetM	tetracycline	antibiotic target protection	0	1

Table S7. Species information and sequence types (STs) of integron-positive isolates.

Strains	Species	ST
KP537	*k. pneumoniae*	1
KP61	*k. pneumoniae*	11
KP389	*k. pneumoniae*	11
KP20	*k. pneumoniae*	11
KP165	*k. pneumoniae*	11
KP16	*k. pneumoniae*	11
KP80	*k. pneumoniae*	11
KP67	*k. pneumoniae*	11
KP599	*k. pneumoniae*	11
KP593	*k. pneumoniae*	11
KP579	*k. pneumoniae*	11
KP565	*k. pneumoniae*	11
KP561	*k. pneumoniae*	11
KP560	*k. pneumoniae*	11
KP558	*k. pneumoniae*	11
KP525	*k. pneumoniae*	11
KP521	*k. pneumoniae*	11
KP510	*k. pneumoniae*	11
KP501	*k. pneumoniae*	11
KP488	*k. pneumoniae*	11
KP480	*k. pneumoniae*	11
KP467	*k. pneumoniae*	11
KP461	*k. pneumoniae*	11
KP453	*k. pneumoniae*	11
KP432	*k. pneumoniae*	11
KP430	*k. pneumoniae*	11
KP428	*k. pneumoniae*	11
KP423	*k. pneumoniae*	11
KP408	*k. pneumoniae*	11
KP404	*k. pneumoniae*	11
KP401	*k. pneumoniae*	11
KP388	*k. pneumoniae*	11
KP385	*k. pneumoniae*	11
KP377	*k. pneumoniae*	11
KP366	*k. pneumoniae*	11
KP360	*k. pneumoniae*	11
KP359	*k. pneumoniae*	11
KP355	*k. pneumoniae*	11
KP354	*k. pneumoniae*	11
KP341	*k. pneumoniae*	11
KP340	*k. pneumoniae*	11
KP339	*k. pneumoniae*	11

KP332	*k. pneumoniae*	11
KP324	*k. pneumoniae*	11
KP315	*k. pneumoniae*	11
KP314	*k. pneumoniae*	11
KP305	*k. pneumoniae*	11
KP288	*k. pneumoniae*	11
KP283	*k. pneumoniae*	11
KP279	*k. pneumoniae*	11
KP268	*k. pneumoniae*	11
KP261	*k. pneumoniae*	11
KP260	*k. pneumoniae*	11
KP257	*k. pneumoniae*	11
KP256	*k. pneumoniae*	11
KP247	*k. pneumoniae*	11
KP243	*k. pneumoniae*	11
KP242	*k. pneumoniae*	11
KP236	*k. pneumoniae*	11
KP226	*k. pneumoniae*	11
KP202	*k. pneumoniae*	11
KP186	*k. pneumoniae*	11
KP166	*k. pneumoniae*	11
KP159	*k. pneumoniae*	11
KP153	*k. pneumoniae*	11
KP149	*k. pneumoniae*	11
KP148	*k. pneumoniae*	11
KP120	*k. pneumoniae*	11
KP117	*k. pneumoniae*	11
KP112	*k. pneumoniae*	11
KP266	*k. pneumoniae*	11
KP598	*k. pneumoniae*	15
KP494	*k. pneumoniae*	23
KP446	*k. pneumoniae*	23
KP491	*k. pneumoniae*	23
KP483	*k. pneumoniae*	23
KP445	*k. pneumoniae*	23
KP387	*k. pneumoniae*	23
KP590	*k. pneumoniae*	23
KP357	*k. pneumoniae*	37
KP431	*k. pneumoniae*	45
KP167	*k. pneumoniae*	45
KP320	*k. pneumoniae*	107
KP365	*k. pneumoniae*	133
KP127	*k. pneumoniae*	340
KP122	*k. pneumoniae*	340

KP443	*k. pneumoniae*	392
KP294	*k. pneumoniae*	423
KP287	*k. pneumoniae*	423
KP450	*k. pneumoniae*	656
KP169	*k. pneumoniae*	685
KP585	*k. pneumoniae*	6026
KP253	*k. pneumoniae*	6254
KP538	*K. variicola* subsp *variicola*	431
KP553	*K. variicola* subsp *variicola*	431
KP307	*K. variicola* subsp *variicola*	429

Table S8. Homologous sequences of IS*26*-Int1 complex resistance regions in the NCBI nucleotide database.

Strains	Coverage (%)	Identity (%)	Accession No.
Klebsiella pneumoniae strain CY814036 plasmid pCY814036-iucA, complete sequence	100	100	CP093152.1
Klebsiella pneumoniae strain 130411-38618 plasmid p130411-38618_1, complete sequence	100	99.99	MK649826.1
Klebsiella pneumoniae strain SCH6109 plasmid pSCH6109-Vir, complete sequence	100	99.95	CP050860.1
Klebsiella pneumoniae strain WCHKP115011 plasmid pVir_115011, complete sequence	100	99.92	CP089955.1
Klebsiella pneumoniae strain S161-2 plasmid pS161-2.3, complete sequence	100	99.91	CP058547.1
Escherichia coli strain STLEFF_47 plasmid unnamed2, complete sequence	99	99.99	CP058876.1
Escherichia coli strain ECNB21-M121 plasmid pNDM-M121, complete sequence	99	99.97	CP083586.1
Klebsiella pneumoniae strain Kp202 plasmid pKp202_1, complete sequence	98	99.96	CP041083.1
Proteus mirabilis strain CCUG 70746 plasmid pPmi70746_1, complete sequence	98	99.96	CP023274.1
Escherichia coli strain AMA566 plasmid pAMA566, complete sequence	98	99.96	MG450360.1
Klebsiella pneumoniae strain AE-2247421-T0 plasmid p2247421-T0_1, complete sequence	98	99.96	CP086448.1
Klebsiella pneumoniae strain AE-2247421-T5-MAC plasmid p2247421-T5-MAC_1, complete sequence	98	99.96	CP086440.1
Klebsiella pneumoniae strain AE-2247421-T5-ESBL plasmid p2247421-T5-ESBL_1, complete sequence	98	99.96	CP086432.1
Klebsiella pneumoniae strain AE-2247421-T20-MAC plasmid p2247421-T20-MAC_1, complete sequence	98	99.96	CP086424.1
Klebsiella pneumoniae strain AE-2247421-T20-ESBL plasmid p2247421-T20-ESBL_1, complete sequence	98	99.96	CP086417.1
Escherichia coli strain Esco-36073cz plasmid pEsco-36073cz, complete sequence	98	99.98	MG252895.1
Klebsiella pneumoniae plasmid IncA/C-LS6, complete sequence	98	99.95	JX442976.1
Providencia stuartii strain 3347685 plasmid p3347685_1, complete sequence	98	99.97	CP071069.1
Aeromonas hydrophila strain 23-C-23 plasmid unnamed, complete sequence	96	99.98	CP038466.1

Aeromonas hydrophila strain WCX23 plasmid unnamed, complete sequence	96	99.98	CP038464.1
Aeromonas hydrophila strain WCX23 plasmid pWCX23_1, complete sequence	96	99.98	CP028419.1
Escherichia coli plasmid pEC11-1b, complete sequence	96	99.97	MT559994.1
Escherichia coli plasmid pEC14-1b, complete sequence	96	99.94	MT559997.1
Klebsiella pneumoniae isolate INF310-sc-2280104 genome assembly, plasmid: 3	95	99.98	LR890256.1
Klebsiella pneumoniae isolate KSB1_5H-sc-2280283 genome assembly, plasmid: 3	95	99.98	LR890502.1
Klebsiella pneumoniae isolate INF298 genome assembly, plasmid: 3	95	99.98	LR890428.1
Vibrio cholerae strain YA00120881 plasmid pYA00120881, complete sequence	95	99.97	MT151380.1
Citrobacter freundii str. U2785 genome assembly, plasmid: 2	94	99.98	LS992184.1
Aeromonas simiae strain A6 chromosome, complete genome	93	99.93	CP040449.1
Providencia stuartii plasmid pMR0211, complete sequence	92	99.96	JN687470.1
Aeromonas hydrophila NUITM-VA1 DNA, complete genome	91	100	AP025277.1
Escherichia coli strain P19_598 plasmid pP19_598a, complete sequence	90	99.94	CP067242.1
Escherichia coli strain GD33 plasmid pNDM33-4, complete sequence	90	99.94	CP076649.1
Yokenella regensburgei strain W13 plasmid pYRW13-125, complete sequence	89	99.88	CP050812.1
Proteus mirabilis strain C55 chromosome, complete genome	89	99.98	CP044436.1
Klebsiella pneumoniae strain 2_GR_12 plasmid IncAC2	89	99.98	CP027055.1
Klebsiella pneumoniae strain 1_GR_13 plasmid IncAC2, complete sequence	89	99.93	CP027043.1
Salmonella sp. strain Sa1735 plasmid pSa1753, complete sequence	88	99.83	MT859309.1
Salmonella enterica subsp. enterica serovar Newport strain VNSEC023 plasmid pCFSAN086837, complete sequence	87	100	CP039438.1
Salmonella enterica subsp. enterica serovar Senftenberg strain CVM N18S0991 plasmid pN18S0991, complete sequence	87	99.96	CP082575.1
Salmonella enterica subsp. enterica serovar Montevideo strain FSIS1607968 plasmid pF18S022, complete sequence	87	99.95	CP082450.1
Salmonella enterica subsp. enterica serovar Heidelberg str. N418 plasmid	87	99.98	CP009409.2

pCFSAN000405_01, complete sequence			
Escherichia coli UMNK88 plasmid pUMNK88, complete sequence	87	99.96	HQ023862.1
Escherichia coli strain cq9 plasmid unnamed3, complete sequence	87	99.93	CP031549.1
Escherichia coli strain 92944 plasmid p92944-TEM, complete sequence	86	100	MG860488.1
Salmonella enterica subsp. enterica serovar Uganda strain CVM 22437 plasmid p22437-1, complete sequence	86	99.96	CP051397.1
Salmonella enterica subsp. enterica serovar Uganda strain CVM 20723 plasmid p20723-1, complete sequence	86	99.96	CP051426.1
Salmonella enterica subsp. enterica serovar Uganda strain CVM 22436 plasmid p22436-1, complete sequence	86	99.96	CP051400.1
Vibrio alginolyticus strain VAS3-1 plasmid pVAS3-1, complete sequence	85	99.93	KU160531.1
Salmonella enterica subsp. enterica serovar Goldcoast strain Sal-5364 plasmid pSal-5364, complete sequence	85	99.95	CP039170.1
Salmonella enterica subsp. enterica serovar Goldcoast strain R18.0877 plasmid pR18.0877_278k, complete sequence	85	99.95	CP037959.1
Salmonella enterica subsp. enterica serovar Agona strain R18.0246 plasmid pR18.0246_278k, complete sequence	85	99.95	CP093412.1
Salmonella enterica subsp. enterica serovar Agona strain R19.0144 plasmid pR19.0144_302k, complete sequence	85	99.95	CP093408.1
Salmonella enterica subsp. enterica serovar Agona strain R21.0464 chromosome, complete genome	85	99.93	CP093402.1
Salmonella enterica subsp. enterica serovar Agona strain SG17-135 plasmid pSG17-135-HI2, complete sequence	85	99.93	CP048776.1
Salmonella enterica subsp. enterica serovar Goldcoast strain R18.1656 plasmid p270k, complete sequence	85	99.93	CP062224.1
Salmonella enterica subsp. enterica serovar Goldcoast strain 5ASAL07 plasmid	85	99.93	CP090134.1

p5ASAL07_294k, complete sequence			
Salmonella enterica subsp. enterica serovar Goldcoast strain 5ASAL05 plasmid p5ASAL05_300k, complete sequence	85	99.91	CP090138.1
Salmonella enterica subsp. enterica serovar Goldcoast strain 5ASAL09 plasmid p5ASAL09_294k, complete sequence	85	99.9	CP090142.1
Shigella dysenteriae strain SWHE2 plasmid unnamed1, complete sequence	85	99.89	CP055064.1
Vibrio parahaemolyticus strain VPS92 plasmid pVPS92-VEB, complete sequence	85	99.91	KU356480.1
Salmonella enterica subsp. arizonae strain S499 plasmid unnamed2, complete sequence	84	99.97	CP082956.1
Vibrio cholerae O139 strain ICDC-211 plasmid pVC211, complete sequence	84	99.94	KY399978.1
Salmonella enterica subsp. enterica serovar London strain HA3-IN1 plasmid pYUHAP1, complete sequence	83	99.95	CP060133.1
Shigella flexneri strain SWHIN_104 plasmid unnamed1, complete sequence	83	99.91	CP055110.1
Shigella flexneri strain SWHIN_107 plasmid unnamed1, complete sequence	83	99.9	CP055100.1
Salmonella enterica subsp. enterica serovar Heidelberg strain CVM N16S074 plasmid pN16S074-1, complete sequence	83	99.98	CP082737.1
Salmonella enterica subsp. enterica serovar Ohio strain FSIS11705518 plasmid pF18S036-1, complete sequence	83	99.97	CP082407.1
Salmonella enterica subsp. enterica serovar Kentucky strain CFSAN007428 isolate N11150 plasmid pCFSAN007428_01, complete sequence	83	99.97	CP009414.2
Klebsiella pneumoniae strain SWHIN_106 plasmid unnamed2, complete sequence	83	100	CP055108.1
Klebsiella pneumoniae strain K-1L plasmid pK-1L-1, complete sequence	83	99.95	CP072461.1
Klebsiella pneumoniae strain b1-2L plasmid pb1-2L-2, complete sequence	83	99.97	CP072458.1
Salmonella enterica subsp. enterica serovar Goldcoast strain R18.1074 plasmid p265k, complete sequence	83	99.92	CP062226.1
Escherichia coli strain RCAD0514 plasmid pRCAD0514EC-1, complete sequence	83	100	CP034107.1
Shigella flexneri strain STLEFF_33 plasmid unnamed1, complete sequence	82	99.96	CP058887.1

Proteus mirabilis strain Pm14C18 plasmid pPm14C18	82	99.97	KU605240.1
Escherichia coli strain 98.1 plasmid p1, complete sequence	82	100	CP059954.1
Escherichia coli strain H3 plasmid A, complete genome	82	99.92	CP010168.1
Salmonella sp. SJTUF14152 chromosome, complete genome	82	99.88	CP064671.1
Escherichia fergusonii strain HNCF11W plasmid pHNCF11W-130kb, complete sequence	82	99.95	CP053046.1
Escherichia coli strain FORC_082 plasmid pFORC82_1, complete sequence	82	99.98	CP026642.1
Shigella flexneri strain STEFF_17 plasmid unnamed1, complete sequence	81	99.99	CP055182.1
Escherichia coli strain GD-33 plasmid pNDM33-1, complete sequence	81	99.99	MN915011.1
Escherichia coli strain TJ-33 plasmid pNDM-TJ33, complete sequence	81	99.99	MN915010.1
Escherichia coli isolate ECCNB12-2 plasmid pTB-nb4, complete sequence	81	99.99	CP033636.1
Escherichia coli strain STEC636 plasmid pSTEC636_1, complete sequence	81	99.99	CP061213.1
Escherichia coli strain GD33 plasmid pNDM33-1, complete sequence	81	99.99	CP076648.1
Salmonella sp. SAL-045 plasmid unnamed1, complete sequence	81	99.99	CP071694.1
Escherichia coli strain ECY44 plasmid pECY44-1, complete sequence	81	99.99	CP069702.1
Escherichia coli strain XH988 plasmid pXH988_1, complete sequence	81	99.97	CP019353.1
Escherichia coli strain TH9F11 plasmid pHNTH9F11-1, complete sequence	81	99.96	CP054192.1
Salmonella enterica subsp. enterica serovar Typhimurium strain S304 plasmid pS304_1, complete sequence	81	99.96	CP061127.1
Klebsiella pneumoniae strain STIN_88 plasmid unnamed1, complete sequence	81	99.96	CP054991.1
Escherichia coli plasmid p16EC-IncN, complete sequence	81	100	MN086778.1
Escherichia fergusonii strain EF20JDJ4045 plasmid pEF45-1, complete sequence	81	99.98	CP086605.1
Salmonella enterica subsp. enterica serovar Derby strain SA1982 plasmid unnamed, complete sequence	81	100	MT513102.1
Escherichia coli strain pV01-18-E02-025-051 plasmid pK18EC051, complete sequence	80	99.95	CP049300.1
Escherichia coli strain EC008 plasmid pEC008, complete sequence	80	99.94	KY748190.1
Escherichia coli strain XH992 plasmid pXH992_2, complete sequence	80	99.94	CP019395.1

Escherichia coli strain GZB8C57M plasmid p8C57-NDM, complete sequence	80	99.94	MT407546.1
Escherichia coli strain 1108 plasmid p1108-NDM, complete sequence	80	99.94	MG825381.1
Escherichia coli strain 2271 plasmid pCTXM-2271, complete sequence	80	99.92	MF589339.1
Salmonella enterica subsp. enterica serovar Typhimurium strain CFSAN007405 isolate 30034 plasmid pCFSAN007405_01, complete sequence	80	99.98	CP009410.2
Salmonella enterica subsp. enterica serovar Newport strain 0307-213, complete genome	80	99.97	CP012599.1
Salmonella enterica subsp. enterica serovar Typhimurium strain CVM 24350 plasmid p24350-1	80	99.97	CP051387.1
Salmonella enterica subsp. enterica serovar Heidelberg plasmid pSH163_135, complete sequence	80	99.97	JN983045.1
Klebsiella pneumoniae plasmid pKC2-1/2a, complete sequence	80	99.95	MT560000.1
Klebsiella pneumoniae plasmid pKC1-1/2a, complete sequence	80	99.95	MT559999.1
Salmonella enterica subsp. enterica serovar Heidelberg plasmid pSH696_135, complete sequence	80	99.89	JN983048.1
Salmonella enterica subsp. enterica serovar Dublin strain N13-01125 plasmid pN13-01125, complete sequence	80	99.97	KX815983.1
Salmonella enterica subsp. enterica serovar Agona strain CVM 21974 plasmid p21794, complete sequence	80	99.95	CP051403.1
Escherichia coli strain SCEC020023 plasmid pOXA10_020023, complete sequence	80	100	CP025944.4

Table S9. Homologous sequences of the class 1 integron *int1-aadA2-qacE1-sul1* in the NCBI nucleotide database.

Strains	Location	Coverage (%)	Identity (%)	Accession No.
Klebsiella pneumoniae strain 16HN-263 chromosome, complete genome	chromosome	100	100	CP045263.1
Klebsiella pneumoniae strain KP18-2079 chromosome, complete genome	chromosome	100	100	CP048933.1
Klebsiella pneumoniae strain KP18-3-8 chromosome, complete genome	chromosome	100	100	CP048430.1
Klebsiella pneumoniae strain 11021 chromosome, complete genome	chromosome	100	100	CP030317.1
Klebsiella pneumoniae strain 283747 chromosome, complete genome	chromosome	100	100	CP030300.1
Klebsiella pneumoniae strain C2414 chromosome, complete genome	chromosome	100	100	CP039819.1
Klebsiella pneumoniae strain C2660 chromosome, complete genome	chromosome	100	100	CP039808.1
Klebsiella pneumoniae strain LSH-KPN148 chromosome, complete genome	chromosome	100	100	CP040122.1
Klebsiella pneumoniae strain KP19-2029 chromosome, complete genome	chromosome	100	100	CP047160.1
Klebsiella pneumoniae strain C1398 chromosome	chromosome	100	100	CP034420.1
Klebsiella pneumoniae strain C789 chromosome, complete genome	chromosome	100	100	CP034415.1
Klebsiella pneumoniae strain KP58 chromosome, complete genome	chromosome	100	100	CP041373.1

Klebsiella pneumoniae strain WCHKP36 chromosome, complete genome	chromosome	100	100	CP028583.2
Klebsiella pneumoniae strain WCHKP8F4 chromosome, complete genome	chromosome	100	100	CP027068.3
Klebsiella pneumoniae subsp. pneumoniae strain SCKP020079 chromosome, complete genome	chromosome	100	100	CP029384.2
Klebsiella pneumoniae strain WCHKP2 chromosome, complete genome	chromosome	100	100	CP028542.3
Klebsiella pneumoniae strain L39_2 chromosome, complete genome	chromosome	100	100	CP033954.1
Klebsiella pneumoniae strain L482 chromosome, complete genome	chromosome	100	100	CP033960.1
Klebsiella pneumoniae strain XJ-K1 chromosome, complete genome	chromosome	100	100	CP032163.1
Klebsiella pneumoniae strain CR-HvKP4 chromosome, complete genome	chromosome	100	100	CP040539.1
Klebsiella pneumoniae strain CR-HvKP5 chromosome, complete genome	chromosome	100	100	CP040545.1
Klebsiella pneumoniae strain CR-HvKP1 chromosome, complete genome	chromosome	100	100	CP040533.1
Klebsiella pneumoniae strain SCKP020009 chromosome, complete genome	chromosome	100	100	CP038002.1
Klebsiella pneumoniae strain WCHKP020037 chromosome, complete genome	chromosome	100	100	CP036371.1
Klebsiella pneumoniae subsp. pneumoniae strain WCHKP015093 chromosome, complete genome	chromosome	100	100	CP036300.1

Klebsiella pneumoniae strain WCHKP020098 chromosome, complete genome	chromosome	100	100	CP036305.1
Klebsiella pneumoniae strain BJCFK909 chromosome, complete genome	chromosome	100	100	CP034123.1
Klebsiella pneumoniae strain 675920 chromosome 675920	chromosome	100	100	CP033242.1
Klebsiella pneumoniae strain F138 chromosome, complete genome	chromosome	100	100	CP026149.1
Klebsiella pneumoniae strain F127 chromosome, complete genome	chromosome	100	100	CP026140.1
Klebsiella pneumoniae strain F77 chromosome, complete genome	chromosome	100	100	CP026136.1
Klebsiella pneumoniae strain F5 chromosome, complete genome	chromosome	100	100	CP026132.1
Klebsiella pneumoniae strain F1 chromosome, complete genome	chromosome	100	100	CP026130.1
Klebsiella pneumoniae strain TVGHCRE225 chromosome, complete genome	chromosome	100	100	CP023722.1
Klebsiella pneumoniae strain L491 chromosome	chromosome	100	100	CP029226.1
Klebsiella pneumoniae strain L388 chromosome	chromosome	100	100	CP029220.1
Klebsiella pneumoniae strain L201 chromosome	chromosome	100	100	CP029216.1
Klebsiella pneumoniae subsp. pneumoniae strain GD4 chromosome, complete genome	chromosome	100	100	CP025951.1
Klebsiella pneumoniae strain F44 chromosome, complete genome	chromosome	100	100	CP025461.1
Klebsiella pneumoniae strain KP69 chromosome, complete genome	chromosome	100	100	CP025456.1
Klebsiella pneumoniae strain FDAARGOS_444 chromosome, complete genome	chromosome	100	100	CP023941.1
Klebsiella pneumoniae strain FDAARGOS_443 chromosome, complete genome	chromosome	100	100	CP023933.1

Klebsiella pneumoniae strain 911021 chromosome, complete genome	chromosome	100	100	CP022882.1
Klebsiella pneumoniae strain SWU01 chromosome, complete genome	chromosome	100	100	CP018454.1
Klebsiella pneumoniae subsp. pneumoniae strain DD01754 chromosome, complete genome	chromosome	100	100	CP087645.1
Klebsiella pneumoniae subsp. pneumoniae strain DD02391 chromosome, complete genome	chromosome	100	100	CP087639.1
Klebsiella pneumoniae subsp. pneumoniae strain DD02297 chromosome, complete genome	chromosome	100	100	CP087634.1
Klebsiella pneumoniae subsp. pneumoniae strain DD02341 chromosome, complete genome	chromosome	100	100	CP087629.1
Klebsiella pneumoniae subsp. pneumoniae strain DD01304 chromosome, complete genome	chromosome	100	100	CP087606.1
Klebsiella pneumoniae strain 21072329 chromosome, complete genome	chromosome	100	100	CP095234.1
Klebsiella pneumoniae strain 21080534 chromosome, complete genome	chromosome	100	100	CP095247.1
Klebsiella pneumoniae strain 21080237 chromosome, complete genome	chromosome	100	100	CP095240.1
Klebsiella pneumoniae strain KP15 chromosome, complete genome	chromosome	100	100	CP087142.1
Klebsiella pneumoniae strain KP16 chromosome, complete genome	chromosome	100	100	CP087146.1
Klebsiella pneumoniae strain KP14 chromosome, complete genome	chromosome	100	100	CP087151.1

Klebsiella pneumoniae subsp. pneumoniae strain WCHKP020039 chromosome, complete genome	chromosome	100	100	CP043344.1
Klebsiella pneumoniae strain WCHKP115038 chromosome, complete genome	chromosome	100	100	CP043602.1
Klebsiella pneumoniae strain WCHKP090050 chromosome, complete genome	chromosome	100	100	CP043369.1
Klebsiella pneumoniae strain WCHKP090045 chromosome, complete genome	chromosome	100	100	CP043365.1
Klebsiella pneumoniae strain FO15 chromosome, complete genome	chromosome	100	100	CP073002.1
Klebsiella pneumoniae strain XH1507 chromosome, complete genome	chromosome	100	100	CP092793.1
Klebsiella pneumoniae strain XH1508 chromosome, complete genome	chromosome	100	100	CP092786.1
Klebsiella pneumoniae strain KP697 chromosome, complete genome	chromosome	100	100	CP066151.1
Klebsiella pneumoniae strain CP19 chromosome, complete genome	chromosome	100	100	CP073351.1
Klebsiella pneumoniae strain kp5152 chromosome, complete genome	chromosome	100	100	CP090462.1
Klebsiella pneumoniae strain F94 chromosome, complete genome	chromosome	100	100	CP090398.1
Klebsiella pneumoniae strain JNKPN26 chromosome, complete genome	chromosome	100	100	CP090203.1
Klebsiella pneumoniae strain C2582 chromosome	chromosome	100	100	CP079208.1
Klebsiella pneumoniae strain C1398 mutant C1 chromosome, complete genome	chromosome	100	100	CP080584.1

Klebsiella pneumoniae strain 50700 chromosome, complete genome	chromosome	100	100	CP088991.1
Klebsiella pneumoniae strain 8695 chromosome, complete genome	chromosome	100	100	CP085889.1
Klebsiella pneumoniae strain KP200731214 chromosome	chromosome	100	100	CP084743.1
Klebsiella pneumoniae strain 1864 chromosome, complete genome	chromosome	100	100	CP084492.1
Klebsiella pneumoniae strain 37 chromosome, complete genome	chromosome	100	100	CP082753.1
Klebsiella pneumoniae strain 36 chromosome, complete genome	chromosome	100	100	CP082759.1
Klebsiella pneumoniae strain 12 chromosome, complete genome	chromosome	100	100	CP082765.1
Klebsiella pneumoniae strain KP19-2196 chromosome, complete genome	chromosome	100	100	CP082041.1
Klebsiella pneumoniae strain KP18-2172 chromosome, complete genome	chromosome	100	100	CP082037.1
Klebsiella pneumoniae strain KP18-2138 chromosome, complete genome	chromosome	100	100	CP082032.1
Klebsiella pneumoniae strain KP18-238 chromosome, complete genome	chromosome	100	100	CP082014.1
Klebsiella pneumoniae strain KP18-41 chromosome, complete genome	chromosome	100	100	CP082010.1
Klebsiella pneumoniae strain KP18-1 chromosome, complete genome	chromosome	100	100	CP082001.1
Klebsiella pneumoniae strain F726925 chromosome, complete genome	chromosome	100	100	CP081820.1
Klebsiella pneumoniae strain KPC-2 chromosome, complete genome	chromosome	100	100	CP078122.1

Klebsiella pneumoniae strain KP-426 chromosome, complete genome	chromosome	100	100	CP080309.1
Klebsiella pneumoniae strain KP-CT77 chromosome, complete genome	chromosome	100	100	CP080303.1
Klebsiella pneumoniae strain KP-C76 chromosome, complete genome	chromosome	100	100	CP080297.1
Klebsiella pneumoniae strain CDI694 chromosome, complete genome	chromosome	100	100	CP077773.1
Klebsiella pneumoniae strain KPCZA02 chromosome, complete genome	chromosome	100	100	CP058226.1
Klebsiella pneumoniae strain KP55 chromosome, complete genome	chromosome	100	100	CP055294.1
Klebsiella pneumoniae strain Kp7224 chromosome	chromosome	100	100	CP074199.1
Klebsiella pneumoniae subsp. pneumoniae strain kpn-hnqyy chromosome, complete genome	chromosome	100	100	CP074116.1
Klebsiella pneumoniae strain 135077 chromosome, complete genome	chromosome	100	100	CP073290.1
Klebsiella pneumoniae strain IR5726 chromosome, complete genome	chromosome	100	100	CP061957.1
Klebsiella pneumoniae strain KPWX136 chromosome, complete genome	chromosome	100	100	CP069170.1
Klebsiella pneumoniae subsp. pneumoniae strain RJBSI76-pV chromosome, complete genome	chromosome	100	100	CP068684.1
Klebsiella pneumoniae strain WCHKP090374 chromosome, complete genome	chromosome	100	100	CP066534.1

Klebsiella pneumoniae strain WCHKP090357 chromosome, complete genome	chromosome	100	100	CP066523.1
Klebsiella pneumoniae strain WCHKP090361 chromosome, complete genome	chromosome	100	100	CP066528.1
Klebsiella pneumoniae strain CRKP78R chromosome, complete genome	chromosome	100	100	CP066254.1
Klebsiella pneumoniae strain CRKP52R chromosome, complete genome	chromosome	100	100	CP066249.1
Klebsiella pneumoniae strain FK 6768 chromosome, complete genome	chromosome	100	100	CP065554.1
Klebsiella pneumoniae subsp. pneumoniae strain WCHKP020120 chromosome, complete genome	chromosome	100	100	CP043357.1
Klebsiella pneumoniae strain JX-CR-hvKP-1 chromosome, complete genome	chromosome	100	100	CP064252.1
Klebsiella pneumoniae strain JX-CR-hvKP-2 chromosome, complete genome	chromosome	100	100	CP064246.1
Klebsiella pneumoniae strain FRPDR chromosome, complete genome	chromosome	100	100	CP063759.1
Klebsiella pneumoniae strain MH9CRKP chromosome, complete genome	chromosome	100	100	CP048412.1
Klebsiella pneumoniae subsp. pneumoniae strain SCKP020143 chromosome, complete genome	chromosome	100	100	CP028548.2
Klebsiella pneumoniae isolate 91eed288-b809-11e8-aae5-3c4a9275d6c8 genome assembly, chromosome: 1	chromosome	100	100	LR596808.1
Klebsiella pneumoniae strain 19PDR22 plasmid p2, complete sequence	plasmid	100	100	CP076548.1

Enterobacter asburiae strain AMA 497 plasmid pOXA436, complete sequence	plasmid	100	100	KY863418.1

Table S10. Homologous sequences of the *bla*$_{IMP-4}$-carrying integron in the NCBI nucleotide database.

Strains	Coverage (%)	Identity (%)	Similarity	Accession No.
K. quasipneumoniae subsp. quasipneumoniae plasmid p2019SCSN059_tmexCD_333k	100%	99.98	99.98	ON169978.1
K. pneumoniae KP294 plasmid pIMP4-KP294,	100%	99.98	99.98	CP083446.1
K. quasipneumoniae SWMUF35 plasmid pA,	100%	99.98	99.98	CP068445.1
K. quasipneumoniae A708 plasmid pA708-1,	100%	99.98	99.98	CP026369.1
K. aerogenes AR_0161 plasmid unnamed,	100%	99.98	99.98	CP028952.1
K. pneumoniae A708 plasmid pA708-IMP,	100%	99.98	99.98	MF344567.1
K. pneumoniae KP1814 plasmid pKP1814-1,	100%	99.98	99.98	KX839207.1
K. michiganensis 7525 plasmid pKOX7525_1,	100%	99.98	99.98	CP065475.1
K. variicola plasmid pFK2020ZBJ35_tmexCD_325k,	100%	99.98	99.98	ON169979.1
E. asburiae AR2284-yvys plasmid pAR2284_1,	100%	99.91	99.91	CP083831.1
P. huaxiensis WCHPr000369 plasmid pIMP4_000369,	99.9	98.901	18784	CP031121.1
C. freundii Cf52 plasmid pCf52,	95.77	94.8123	219342	KY887592.1
C. freundii Cf53 plasmid pCf53,	95.77	94.8123	200459	KY887593.1
A. caviae SCLZS52 plasmid pIMP_SCLZS52,	99.94	89.946	113450	CP091177.1
E. cloacae EC62 plasmid pIMP-4-EC62,	99.92	89.928	314351	MH829594.1
A. caviae NY4625 plasmid pNY4625-IMP,	99.92	89.928	111644	MN629346.1
A. caviae NY4622 plasmid pNY4622-IMP,	99.92	89.928	113608	MN961666.1
K. pneumoniae subsp. pneumoniae KP67 plasmid p67_1,	99.9	89.91	318780	CP101561.1
E. cloacae ECL-13-46 plasmid pIMP-KP-13-9, partial sequence	98.96	89.064	46244	CP068240.1
E. hormaechei NJGLYY90-CR plasmid pECL-90-2,	99.95	82.9585	348891	CP061746.1

K. variicola strain 13450 plasmid p13450-1,	99.95	82.9585	344479	CP026014.1
K. pneumoniae 19051 plasmid p19051-IMP,	99.95	82.9585	316843	MF344565.1
K. pneumoniae 13450 plasmid p13450-IMP,	99.95	82.9585	344478	MF344564.1
K. pneumoniae 11219 plasmid p11219-IMP,	99.95	82.9585	319852	MF344561.1
R. ornithinolytica Ro24724 plasmid pRo24724,	99.95	82.9585	446611	CP021328.1
K. pneumoniae C1672 plasmid pRes_C1672	99.95	82.9585	329021	CP073918.1
E. cloacae RJ702 plasmid pIMP26,	99.92	82.9336	329420	MH399264.1
E. cloacae complex sp. strain ECL60 plasmid pIMP26-ECL60,	99.92	82.9336	320374	CP083235.1
K. pneumoniae KP1572 plasmid pIMP1572,	99.92	82.9336	142993	MH464586.1
S. marcescens YL4 plasmid p1,	99.92	82.9336	316459	CP083755.1
E. cloacae complex sp. ECL405 plasmid unnamed1,	99.9	82.917	362923	CP091493.1
E. hormaechei L51 plasmid pEHZJ1,	99.9	79.92	343918	CP033103.1
